# Transposon-mediated telomere destabilization: a driver of genome evolution in the blast fungus

**DOI:** 10.1093/nar/gkaa287

**Published:** 2020-06-19

**Authors:** Mostafa Rahnama, Olga Novikova, John H Starnes, Shouan Zhang, Li Chen, Mark L Farman

**Affiliations:** Department of Plant Pathology, University of Kentucky, 1405 Veteran's Dr., Lexington, KY 40546, USA; Department of Plant Pathology, University of Kentucky, 1405 Veteran's Dr., Lexington, KY 40546, USA; Department of Plant Pathology, University of Kentucky, 1405 Veteran's Dr., Lexington, KY 40546, USA; Department of Plant Pathology, University of Kentucky, 1405 Veteran's Dr., Lexington, KY 40546, USA; Department of Plant Pathology, University of Kentucky, 1405 Veteran's Dr., Lexington, KY 40546, USA; Department of Plant Pathology, University of Kentucky, 1405 Veteran's Dr., Lexington, KY 40546, USA

## Abstract

The fungus *Magnaporthe oryzae* causes devastating diseases of crops, including rice and wheat, and in various grasses. Strains from ryegrasses have highly unstable chromosome ends that undergo frequent rearrangements, and this has been associated with the presence of retrotransposons (*Magnaporthe oryzae* Telomeric Retrotransposons—MoTeRs) inserted in the telomeres. The objective of the present study was to determine the mechanisms by which MoTeRs promote telomere instability. Targeted cloning, mapping, and sequencing of parental and novel telomeric restriction fragments (TRFs), along with MinION sequencing of genomic DNA allowed us to document the precise molecular alterations underlying 109 newly-formed TRFs. These included truncations of subterminal rDNA sequences; acquisition of MoTeR insertions by ‘plain’ telomeres; insertion of the MAGGY retrotransposons into MoTeR arrays; MoTeR-independent expansion and contraction of subtelomeric tandem repeats; and a variety of rearrangements initiated through breaks in interstitial telomere tracts that are generated during MoTeR integration. Overall, we estimate that alterations occurred in approximately sixty percent of chromosomes (one in three telomeres) analyzed. Most importantly, we describe an entirely new mechanism by which transposons can promote genomic alterations at exceptionally high frequencies, and in a manner that can promote genome evolution while minimizing collateral damage to overall chromosome architecture and function.

## INTRODUCTION

*Magnaporthe oryzae* (synonymous with *Pyricularia oryzae*) is an ascomycete fungus that causes blast disease in rice, wheat and other crops; and is also responsible for leaf spot diseases of a variety of turf and pasture grasses, including annual ryegrass (1,2), perennial ryegrass (3), tall fescue and St. Augustinegrass (4). Most *M. oryzae* strains exhibit host specificity, being capable of infecting only a very small number of host genera and/or species (5,6). Additional specificity exists at the sub-species level, with rice, wheat and foxtail pathogens being compatible with some cultivars of their respective host species, and not others (7–9). Cultivar specificity has been intensively studied because it is the main foundation upon which plants are bred for blast resistance. Unfortunately, cultivar specificity often breaks down due to a high degree of pathogenic variability within the fungus (10,11). Studies at the molecular level have shown that *M. oryzae* escapes host and cultivar recognition through the mutation (or loss) of genes that code for proteins that are secreted during infection (12,13). These proteins would normally trigger resistance in host plants that contain the corresponding resistance receptors and, for this reason, they are termed ‘avirulence’ effectors. Interestingly, many avirulence genes exhibit a high degree of genetic instability and a large proportion of them (∼50%) map very close to telomeres (14).

Telomeres are the sequences that constitute the ends of linear chromosomes and in most organisms comprise short, tandem repeats—(TTAGGG)_*n*_ in most fungi (incl. *M. oryzae*) and animals (15); and (TTTAGGG)_*n*_ in plants (16). In many organisms, the regions immediately adjacent to the telomeres contain sequences that are duplicated at different chromosome ends—effectively creating a defined subtelomeric domain (17). The distal subtelomere regions usually contain a variety of short, tandem repeat motifs (18–23), and the proximal portions often harbor families of genes which are associated with niche adaptation (24–30). Because the chromosome ends are the most dynamic regions of the genome—often undergoing spontaneous rearrangements (29,31–36), and experiencing accelerated mutation (37–39), they tend to exhibit much higher levels of polymorphism than the genome interior (37,38,40–46). Additionally, genes near telomeres can be subject to stochastic epigenetic regulation (29,32,47–50). Accordingly, the genes that reside near to telomeres benefit from the enhanced evolutionary and adaptive potential afforded by these behaviors (31,35,51–53).

Previously, we reported that telomeric restriction fragments (TRFs) in *M. oryzae* strains from perennial ryegrass are unusually polymorphic when compared to internal chromosomal regions (41). This contrasts with the telomeres of strains from rice, which are remarkably stable by comparison (54). Characterization of unstable telomeres revealed evidence that this polymorphism is due to frequent, spontaneous rearrangements at the chromosome ends and that this instability is associated with the presence of non-LTR retrotransposons embedded within the telomere repeats (54). These transposons are generally lacking from *M. oryzae* strains with stable telomeres (41,54,55). Two related retroelements were identified. The first, MoTeR1, is 5 kb in length and codes for a predicted reverse transcriptase (RT) enzyme that exhibits similarity to RTs encoded by the SLACS retrotransposon from *Giardia lambliae* (56), and CRE1 from *Crithidia faciculata* (57,58). These latter elements are site-specific transposons that insert specifically into splice leader sequence genes, using a restriction enzyme-like endonuclease (REL-ENDO). The MoTeR1 RT contains a putative REL-ENDO domain and possesses an extensive run of telomere-like sequence, TTCGGG(TTTGGG)n, at its ' terminus (54), which leads us to suspect that MoTeR1 is a site-specific transposon that targets telomere repeats in a similar manner to the TRAS and SART retrotransposons in *Bombyx mori* (59).

MoTeR2 shares the same 5′ (860 bp) and 3′ (77 bp) terminal sequences with MoTeR1. However, in MoTeR2, the RT coding region is replaced with an unrelated 786 bp sequence with no obvious function (54). Thus, MoTeR2 is likely a defective element and, if mobile, probably uses the MoTeR1 RT for its transposition. MoTeR1 and MoTeR2 sequences can exist in single copy in a given telomere, or in homogeneous, or heterogeneous arrays. When they occur in tandem, adjacent elements are always arranged in a head to tail orientation, and are separated by a (TTAGGG)_*n*_ tract ranging in length from one half of a repeat unit to more than 20 (54).

Transposons have major impacts on the organization and evolution of genomes (60). Their abundance is a primary determinant of genome size (61,62) and they are responsible for promoting genome change (63,64). Indeed, it is this latter property that led to their initial discovery (65). Aside from simple gene inactivation that results from transposon integration (66), there is the potential for much wider-ranging genomic alterations, including translocations, deletions, segmental duplications and inversions (67–70). Such rearrangements can occur as a result of aberrant transposition (71,72) or, after the fact, through ectopic recombination between dispersed transposon copies (64). Given that MoTeR1 bears all the hallmarks of a site-specific transposon, we hypothesized that the abundant rearrangements observed during vegetative growth *in vitro* and *in planta* (54) might result from frequent transposition events. To test this idea, we first employed shotgun and targeted cloning to characterize a number of newly-formed TRFs, along with the respective chromosome ends in the progenitor strain. Next, we used MinION sequencing to uncover a large number of cryptic telomere alterations. Together, these efforts allowed us to document a wide range of molecular mechanisms giving rise to >100 telomere rearrangements in *M. oryzae;* and to document a novel mechanism by which transposon insertions can promote genome rearrangements.

## MATERIALS AND METHODS

### Passaging the fungus through plants

Two experiments were performed in which the fungus was serially passaged through plants two times, with no artificial culture in between (Supplementary Figure S1). For experiment 1, strain LpKY97 was reactivated from a stock culture by placing on oatmeal agar. Spores were harvested by flooding the plate with a 0.2% gelatin solution and sprayed on leaves of the annual ryegrass cultivar Gulf. For experiment 2, a single spore was isolated from the original LpKY97 culture and used to establish a single spore (SS) culture on oatmeal agar. A spore suspension was made and sprayed on leaves of the perennial ryegrass cultivar Linn. Seven days later, after disease symptoms had appeared, leaves with visible lesions were removed, placed in a moist chamber and incubated overnight at room temperature for sporulation to occur (∼25°). The spores from this second generation were harvested by flooding the plate with a 0.2% gelatin solution and a small aliquot was streaked on water agar, to establish second generation SS cultures. The remainder was used to re-inoculate plants and the process was repeated to generate a third SS generation. Finally, select third generation SS cultures were re-cultured on oatmeal agar and up to 20 single spores were collected and used to generate fourth generation cultures. The various cultures were named according to the following scheme: <experiment#>SS<single-spore-IDs#> (e.g. 2G4SS1–10 indicates experiment 2, fourth-generation spore culture #10, derived from third-generation spore culture #1).

### Generation of single spore isolates from fungus grown on oatmeal agar plates

For experiment #3, a single spore of strain LpKY97-1 was inoculated at the very edge of a Petri dish containing complete medium agar (CMA). This medium allows rapid growth but suppresses sporulation which ensures that the fungus undergoes a maximal number of nuclear divisions in reaching the other side of the plate - instead of jumping via spores. The mycelium was allowed to grow to the opposite side of the plate, at which point a plug containing the mycelial front was excised and used to inoculate a second CMA plate. After the mycelium had grown to the other side of the second plate, a plug containing the mycelial front was placed on oatmeal agar to induce sporulation. Spores were then harvested by gently brushing a sealed Pasteur pipette over the culture and the pipette tip was then streaked across water agar plates. After overnight incubation at room temperature, over 250 germinated spores were individually isolated and used to start a set of ‘3G3’ SS cultures.

### Shotgun cloning of telomeric restriction fragments

Genomic DNA was purified using a standard procedure (54) and polysaccharides were subsequently removed using differential ethanol precipitation (73). Approximately 5 μg of polysaccharide-reduced, undigested genomic DNA was end-repaired using the End-It™ kit (Epicentre Technologies, Madison, WI, USA). The enzyme was removed by phenol:chloroform:isoamyl alcohol (PCI, 25:24:1) extraction (2×), followed by chloroform:IAA (CI, 24:1) extraction (1×) and the DNA was then ethanol-precipitated, rinsed with 70% ethanol, dried and re-dissolved in 9 μl of 1× ligation buffer (New England Biolabs, Ipswich, MA, USA). To this was added: 1 μl of *Eco*RV-digested pBlueScript KS II^+^ (∼100 ng) that had been treated with calf intestinal phosphatase (Promega Corp., Madison, WI, USA), and 0.1 μl of T4 DNA ligase (NEB). Ligation was performed overnight at 12°C. The ligase was killed by heating at 65°C for 10 min and then 5 μl of 10× reaction buffer 3 (NEB), 34 μl of H_2_O and 1 μl of *Pst*I (NEB) were added and restriction digestion was performed at 37°C for 4 h. After digestion, the restriction enzyme was removed by PCI and CI extraction and the DNA was ethanol-precipitated. The DNA pellet was re-dissolved in 50 μl of 1× ligation buffer, 0.1 μl (40 u) of ligase (NEB) was added and the reaction was allowed to proceed at 12°C overnight. Finally, the DNA was ethanol-precipitated and re-dissolved in 20 μl of TE. The ligated DNA sample was used to transform electrocompetent *Escherichia coli* cells (EPI300, Epicentre), using 1 μl of ligated DNA solution per transformation. Telomere-positive clones were identified by colony blotting using a published procedure (74,75).

### Targeted cloning of individual telomeric restriction fragments

The method used to clone specific TRFs through enrichment of terminal sequences has been described in detail elsewhere (74). Briefly, polysaccharide-depleted, genomic DNA samples (∼2 μg) were end-repaired using the End-it kit from Epicentre Technologies (Madison, WI, USA). The enzymes were inactivated and removed using PCI (2×) and CI (1×) extraction and the DNA was ethanol-precipitated and rinsed in 70% ethanol. The DNA was then dissolved in 1× restriction enzyme buffer and digested with *Pst*I for 4 h before being fractionated by gel electrophoresis. Gel slices containing DNA molecules in size ranges spanning the target fragments were excised and the DNA was extracted using the QIAquick kit (Qiagen, Valencia, CA, USA) and eluted in 25 μl elution buffer. The purified fragments (3.5 μl, ∼100 ng) were ligated to *Pst*I + *Eco*RV-digested, CIP-treated pBlueScript KS II^+^ (∼100 ng) in a reaction volume of 5 μl. One microliter of the reaction mix was used to transform ultra-competent EPI300 cells (Epicentre). Telomere-positive clones were identified by colony blotting.

### Southern blotting and hybridization

The methods used for Southern blotting and generation of telomere probes have been described previously (54). Individual telomeres and their corresponding telomeric restriction fragments (TRFs) were named according to their correspondence with the telomeres in the reference genome for strain B71 (76). Telomeres on supernumary (mini- chromosomes) were labeled using A, B, C and D identifiers, with the two mini-chromosomes being labeled 1 and 2 according to their sizes (largest first). Newly-formed telomeres/TRFs that could not be associated with specific telomeres retained the lowercase a, b, c etc. identifiers that were initially assigned to the novel TRFs.

Telomere-linked probes (TLPs) were developed by sequencing the cloned TRFs using T3 and T7 primers, and a primer reading out from the MoTeR 3′ end (MoTeRnR). Where possible, sequences adjacent to multi-MoTeR arrays were acquired using 3′ end-proximal primers that were specific to either MoTeR1 or MoTeR2. Chromosomal sequences adjacent to the MoTeR and the non-telomeric end of the TRF insert were compared with known *M. oryzae* repeats, and primers were designed to amplify single-copy (or low-copy) probes. Primer sequences are listed in Supplementary Table S1.

### MinION sequencing and analysis

Between 2–5 μg of DNA, extracted as described above, was further purified using the MagAttract kit (Qiagen). Sequence-ready libraries were then prepared from 500 ng to 1 μg of intact (non-sheared) DNA using the SQK-LSK109 Ligation Sequencing Kit (Oxford MinION Technologies, Oxford, UK) according to the manufacturer's instructions. Sequence data were acquired on MinION flow cells for between 16 and 24 h. Raw data in fast5 format were converted to fastq using Guppy (77) and individual strain assemblies were generated using canu (78) with default settings. To generate a high quality, base reference for strain LpKY97, a hybrid assembly was created with data from three descendant progeny, using only reads that were 8 kb in length, or longer and single nucleotide errors were corrected using Illumina sequence data, as described in Rahnama *et al.* (submitted). Telomere rearrangements were identified by aligning individual reads to the individual and merged reference genomes using minimap2 (79), followed by manual inspection of the alignments in the Integrative Genomics Viewer (80). Specifically, we examined only those alignments where the reads extended from the unique, telomere adjacent region all the way to the telomere. In this case, we required that the reads contained 10–30 TTAGGG repeats that extended to an end of the sequenced molecule (with an allowance for up to ∼30 nt of sequencing adaptor that was present at the ends of some reads).

## RESULTS AND DISCUSSION

### Terminology

#### Telomeres

The sequences that comprise the chromosome ends—in this case, repeats of the hexanucleotide motif, CCCTAA/TTAGGG.

#### Telomere-adjacent sequences

The sequences immediately subtending the telomeric repeats.

#### Subtelomeres

A term reserved for specific domains that comprise sequences duplicated at multiple chromosome ends, and not usually found elsewhere in the genome.

#### Subterminal sequences/regions

Sequences/regions near the chromosome ends that do not qualify as ‘subtelomeres’, either because they are unique, or they comprise sequences duplicated throughout the genome. Arbitrarily, we define the subterminal regions as being within 100 kb from the chromosome ends.

#### Chromosome-unique sequence

Sequence from a subterminal region that uniquely identifies it as coming from a specific chromosome end.

### Identification of *M. oryzae* cultures with altered telomere ‘fingerprints’

**Figure 1.**
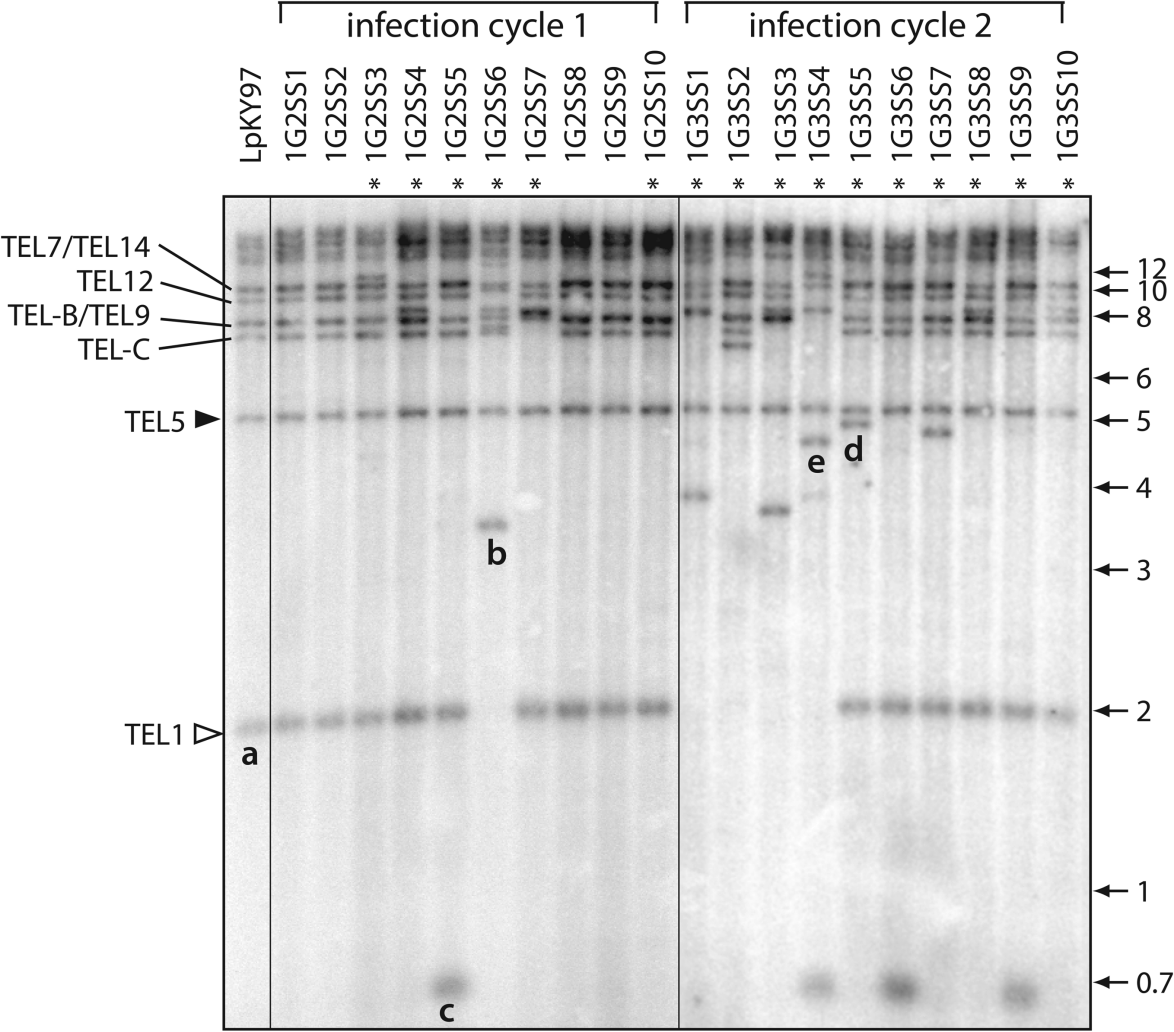
Characterization of rearranged telomeric restriction fragments (TRFs) formed during growth *in planta*. (**A**) Telomeric restriction profiles of single spore isolates from successive rounds of plant infection. DNA samples were digested with *Pst*I, fractionated by gel electrophoresis, blotted to a membrane and hybridized with a telomere probe. TRFs are labeled based on their chromosome locations in the *M. oryzae* B71 reference genome. Asterisks mark lanes with telomere profiles that are different from the parent culture. An open arrowhead marks the highly unstable ‘rDNA’ telomere (TEL1). A closed arrowhead marks a relatively stable telomere that rarely exhibited rearrangement (TEL5). Telomeres on mini-chromosomes 1 and 2 are labeled TEL-B and TEL-C, respectively. Fragments that were successfully cloned and characterized are labeled ‘a’ through ‘e’.

To explore the molecular events responsible for the frequent telomere rearrangements in MoTeR-containing strains, we grew the fungus on culture media and *in planta*, and established a large collection of isolates containing novel telomeric restriction fragments (TRFs). The first set of 20 single-spore isolates came from an experiment in which strain, LpKY97, was passaged through annual ryegrass plants for two successive generations (Supplementary Figure S1). Six of 10 isolates from infection cycle 1 and 10 of 10 from cycle 2 exhibited novel telomere profiles compared with the original parental strain (Figure 1). Isolates containing novel TRFs were selected for further investigation.

First, we used a shotgun strategy to clone and sequence *Pst*I TRFs from the single spore cultures exhibiting telomere alterations. In parallel, we attempted to clone *Pst*I TRFs from the original parent strain, LpKY97. Consistent with their terminal position on the chromosome, all of the cloned TRFs contained telomere repeats ligated directly to the *Eco*RV half-site in the vector, with the C-rich strand reading in the 5′ to 3′ direction.

### ‘rDNA telomere’ truncations

**Figure 2.**
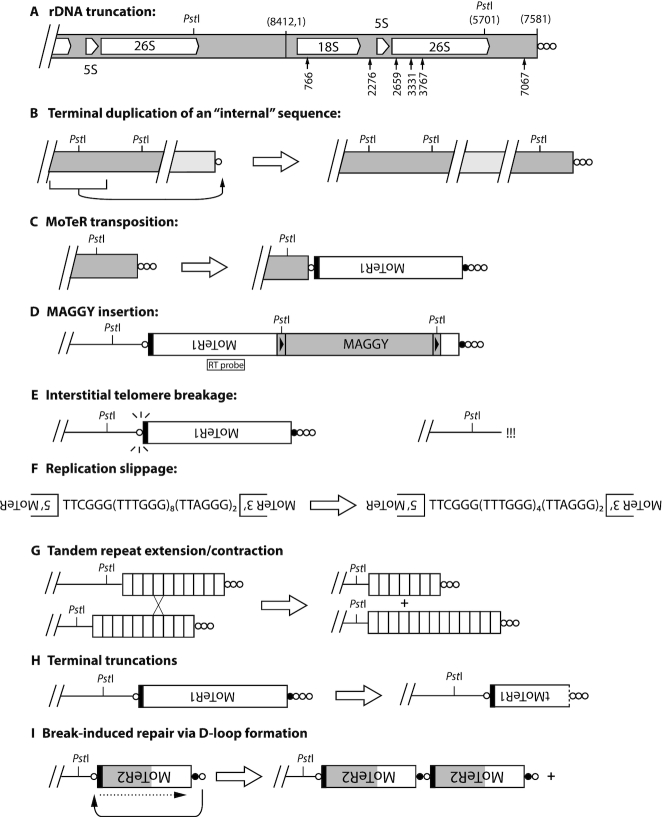
Mechanisms giving rise to rearranged TRFs. (**A**) *Truncations of the ribosomal DNA array* (TEL1). The positions of 18S, 5S and 26S rRNA genes are shown, along with the positions of the two most distal *Pst*I restriction sites. The telomere repeats are denoted as circles with each circle representing 1 to 10 copies of TTAGGG–(TTAGGG)_1–10_. Relevant nucleotide coordinates are provided in parentheses above the chromosome (based on the rDNA reference sequence in NCBI, accession #AB026819.1). The most distal *Pst*I site is at position 5701, which lies ∼2.05 kb from the chromosome end in LpKY97. rDNA TRF truncations were identified by targeted cloning of novel restriction fragments, and by inspection of Illumina and MinION sequence reads. Arrows show positions of telomere addition in truncated variants that were successfully cloned. (**B**) *Duplication of an internal sequence at a chromosome end*—presumably initiated when a chromosome end is effectively de-capped (≤10 TTAGGG repeats). (**C**) *MoTeR acquisition by plain telomeres*. Regardless, of whether it occurs through recombination, BIR, or transposition, MoTeR acquisition results in the internalization of telomere repeats, creating interstitial telomeres. Note: the black shading at the MoTeR1 3′ end represents 3′ sequence shared with MoTeR2. The MoTeR1 label is inverted to illustrate that telomeric elements are always oriented with their 5′ ends nearest to the chromosome end. White circles = (TTAGGG)_1–10_ repeats; black = TTCGGG(TTTGGG)_5–8_. (**D**) *MAGGY insertion into MoTeR sequences*. Shown is a full-length MAGGY element inserted in an inverted orientation relative to MoTeR1. This diagram shows MAGGY inserted distal to the MoTeR1 RT probe. Restriction sites in the MAGGY LTRs causes the probe to hybridize with non-telomeric (i.e. internal fragments) in *Pst*I digests. (**E**) *Interstitial telomere breakage*. Internal TTAGGG tracts >3 repeat units are prone to breakage, generating recombinogenic ends that can be repaired by various processes operating on double-stranded breaks. The pathway utilized likely depends on the nature of the sequences at the exposed ends. (**F**) *Replication slippage*. Polymerase pausing and dissociation leads to skipping forward/backward in a short tandem repeat array, resulting in contraction/expansion of the array. (**G**) *Subtelomeric tandem repeat (STR) contraction/expansion*. The characteristic feature of subtelomeric repeat contraction/expansion is preservation of the telomere junction. Array length changes can occur via unequal chromatid exchange (shown here) or gene conversion, with contractions also being possible as a result of intrachromatid recombination. (**H**) *MoTeR truncations*. These relatively rare occurrences involve degradation of MoTeR sequences, followed by *de novo* telomere formation (likely through telomerase action). MoTeRs with a dotted line at the 5′ borders represent truncated elements (tMoTeRs), and the truncation position is noted in parentheses. (**I**) *D-loop formation followed by BIR*. Uncapped telomere/MoTeR sequences in a multi-element array potentially can invade homologous sequences in proximal elements and initiate break-induced replication. The ‘+’ symbol indicates that the process can occur in an iterative fashion, generating multi-element extensions. The black shading represents the 3′ border sequences common to MoTeR1 and 2, while the gray shading represents sequence unique to MoTeR2.

In most SS cultures, the smallest fragment hybridizing to the telomere probe (Figure 1, band a) was the ‘rDNA’ telomere (TEL1) which comprises the terminal portion of the tandem ribosomal DNA repeat array with the telomere attached 1567 bp downstream of the 26S rRNA gene (nucleotide position 7581 in the *M. oryzae* rDNA reference sequence, NCBI #AB026818) (Figure 2A). Thirteen of 39 SS progeny recovery from *in planta* passaging lacked the 2 kb fragment (1G2SS6, 1G3SS1, 2, 3 and 4, Figure 1; 2G3SS4, 5, 6, 8 and 9, Supplementary Figure S2; and 3G1SS56, data not shown). Cloning and sequencing of rDNA TRFs from six of these progeny revealed structures consistent with simple terminal truncations, followed by *de novo* telomere formation (Figure 2A), and an additional 20 truncations were detected among Illumina and MinION sequencing reads for representative SS cultures (Supplementary Table S2). Nineteen of 24 *de novo* telomere formation events occurred via extension of short (1–4 nucleotide) telomere ‘seeds’ at the DNA ends (Supplementary Table S2). One culture (1G3SS4) experienced more a complex TEL1 rearrangement (see below).

### Terminal duplication of ‘internal’ sequences

**Figure 3.**
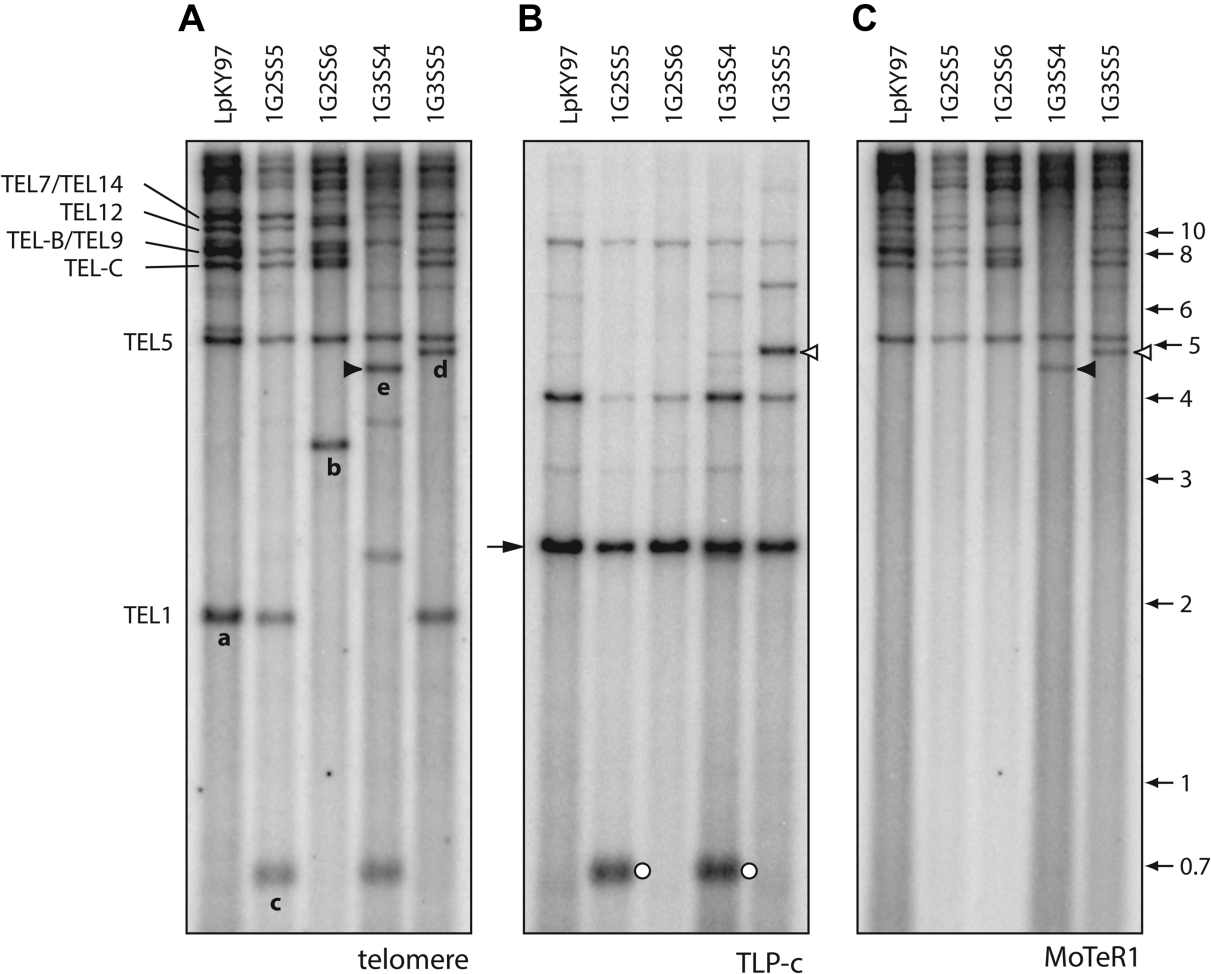
Identification of putative MoTeR1 transposition events. The three panels show selected single spore DNA samples from the population shown in Figure 1. Panels A, B and C were hybridized with the telomere, TLP-c and MoTeR1 probes, respectively. (**A**) Novel TRFs that have been characterized are labeled ‘a’ through ‘e.’ (**B**) Re-probing of the blot with TLP-c produced a major signal in all lanes at a position corresponding to a molecular size of ∼2.5 kb. As expected, the probe hybridized to novel TRF-c, but also appeared to hybridize with novel TRF ‘d’ (open arrowheads). (**C**) The same blot was re-probed with the MoTeR1 probe. This also produced a hybridization signal at a position corresponding to TRF-d, implying that TRF-c had acquired a MoTeR1 insertion. Note that the MoTeR1 probe also hybridized to TRF-e (closed arrowheads)—signaling the occurrence of a second putative transposition event.

Four SS progeny (1G2SS5, 1G3SS4, 1G3SS6 and 1G3SS9) exhibited a 700 bp TRF that was not present in the original parent isolate (Figures 1 and 3A). To determine the origin of this fragment, we cloned and sequenced it, and developed a telomere-linked probe (‘TLP-c’; note: this fragment is unrelated to TEL-C) that was used to re-probe the Southern blot shown in Figure 3A. As expected, the probe hybridized to the TRF-c band (Figure 3B, white circles) but, in the parental strain, the strongest signal came from a ∼2.5 kb fragment that was non-telomeric (cf. Figure 3A). A BLAST search of a chromosome-level reference sequence for LpKY97–1 revealed that the orthologous parental locus resides ∼157 kb from TEL5, while the weaker signals come from short, divergent sequence matches elsewhere in the genome. The conservation of the parental band in the cultures with TRF-c, along with densitometric assessment of the parental band intensities, indicated that the parental band was still present in isolates and, hence, the novel TRF arose via duplication of the internal sequence at a chromosome end (see Figure 2B). Given the unlikelihood that this rearrangement occurred more than once, we conclude that the four SSs inherited a single, original variant during clonal propagation *in planta*.

### Acquisition of MoTeR1 by ‘plain’ telomeres

In culture 1G3SS5, the TLP-c probe hybridized to another telomeric fragment that was not present in the parental culture (see white arrowhead in Figure 3B) and which corresponded to the novel TRF designated ‘d’ (Figures 1 and 3A). Considering it unlikely that the same locus had undergone a second terminal duplication, we surmised that this TRF might have arisen through MoTeR transposition into the newly-formed TEL-c (see Figure 2C). To test this idea, we re-hybridized the membrane sequentially with probes derived from MoTeR1 and MoTeR2. Consistent with our prediction, TRF-d co-hybridized with the MoTeR1 probe (Figure 3C, white arrowhead). The TRF was isolated via targeted cloning (see Methods) and sequencing showed the newly-formed telomere had acquired a truncated MoTeR1 (‘tMoTeR1’) insertion that was missing 783 bp from its 5′ end (Supplementary Figure S3A.i).

Figure 3C shows that the MoTeR1 RT probe also bound to a novel TRF in isolate 1G3SS4 (black arrowhead). Considering that MoTeR1 lacks *Pst*I restriction sites and insertion into a telomere should therefore cause the corresponding TRF size to increase, we reasoned that the novel band was likely derived from TEL1 - the only parental TRF that is smaller; and which was conspicuously absent in the 1G3SS4 culture (Figures 1 and 3A). The novel TRF was isolated via targeted cloning, and sequencing revealed a truncated MoTeR1 (missing 2,251 nt from the 5 ' end) inserted in the rDNA telomere, and positioned just one TTAGGG repeat unit away from the junction between the telomere and the rDNA sequences (Supplementary Figure S3B).

TEL5, which was relatively stable, resided on a 5.7 kb *Pst*I fragment and harbored a single full-length MoTeR1 insertion in the telomere (Supplementary Figure S3C.i; note: telomeric fragments consistently showed slightly retarded migration relative to non-telomeric fragments of the same length). This fragment was lacking in 2G3SS4 (Supplementary Figure S2) and sequential re-probing with TLP5 and MoTeR sequences revealed that the fragment had increased in size by ∼1 kb (Supplementary Figure S2, band ‘f’), and now hybridized with both the MoTeR1 and MoTeR2 probes (results not shown). Targeted cloning and sequencing of the new fragment, as well as MinION sequencing of a related SS culture, 2G4SS4–20 (SS4–20, see below), revealed two truncated MoTeR2 (tMoTeR2) insertions distal to the original MoTeR1 (Supplementary Figure S3C.i). Again, identically truncated elements were not found among Illumina sequencing reads acquired from the original parent, or MinION reads for three other SS isolates, which makes it unlikely that these elements were simply acquired from other chromosome ends via break-induced repair, or gene conversion. On the other hand, rearrangements detected at TEL7 (Supplementary Figure S4B.ii&iv) also involved acquisition of MoTeR copies but, in these cases, the elements were clearly duplicates of sequences found at TEL-D (Supplementary Figure S5). A number of additional rearrangements consistent with MoTeR transposition were identified in TELs 8, B, and C (Supplementary Figures S6A, S7 and S8).

### Transposition of the MAGGY LTR retrotransposon into MoTeR sequences

Among the first newly-formed TRFs isolated using the targeted approach were three that contained telomere repeats linked to the MoTeR 5′ terminus at one end of the insert and sequences from the long terminal repeat (LTR) of the MAGGY retrotransposon at the other (Supplementary Figure S9; Supplementary Table S3). MAGGY contains one *Pst*I site in each LTR and, therefore, insertion of this element into a MoTeR array (which lacks *Pst*I sites) almost invariably causes the size of the terminal *Pst*I fragment to decrease (see Figure 2D). Cultures 3G1SS56 and 3G1SS145 inherited the same MAGGY insertion, which occurred in the penultimate MoTeR element, relative to the telomere. However, because the integration was in the 5′ region conserved between MoTeR1 and MoTeR2, the identity of the target MoTeR (1 versus 2) was undetermined. By comparing MinION genome assemblies for three different SS isolates, we could infer that the parental LpKY97 strain contained only one MoTeR element with a MAGGY insertion, which occurred in an extended MoTeR array in TEL-D (Supplementary Figure S5). In turn, this confirmed that the three cloned copies represented new integration events.

**Figure 4.**
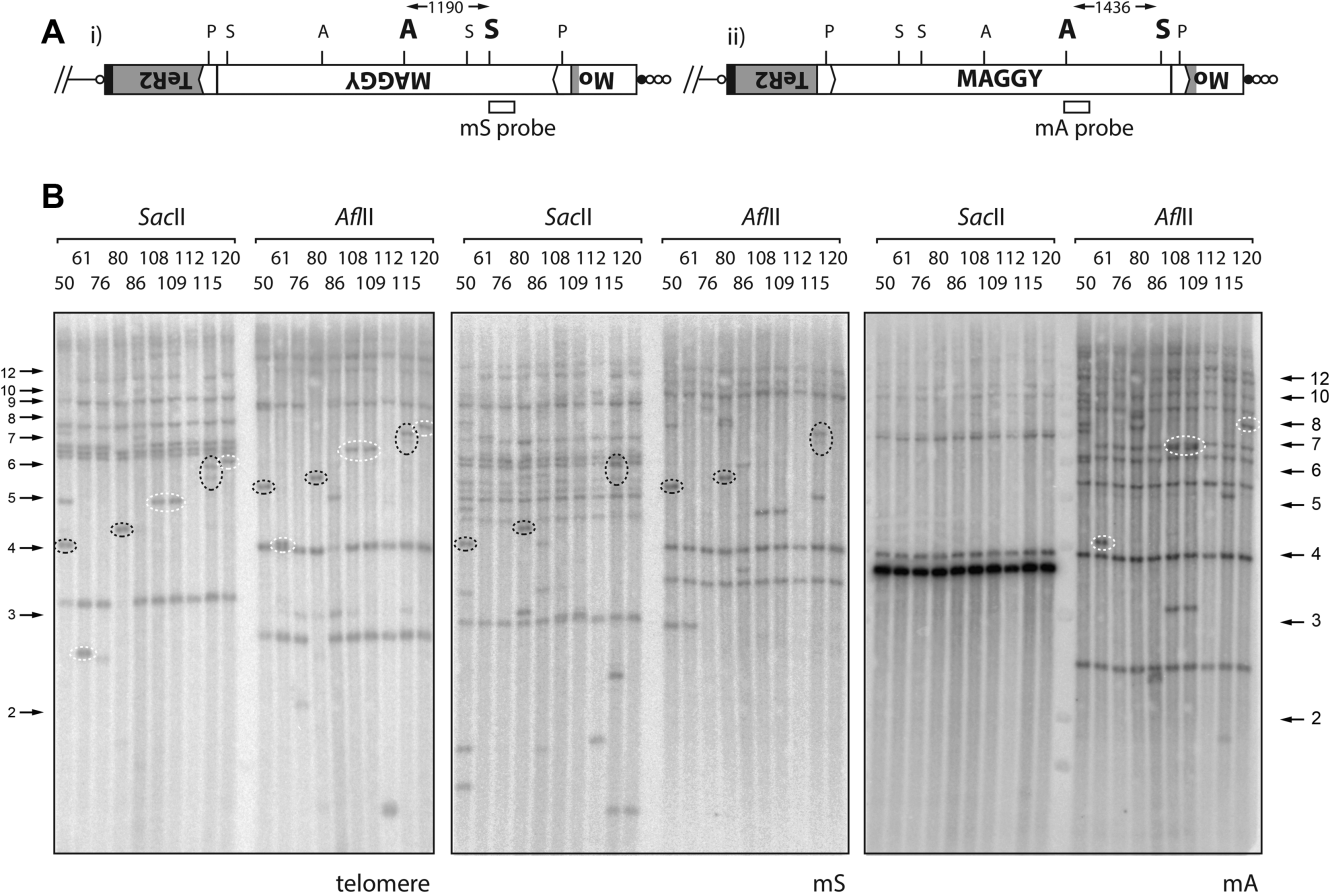
Detection of new MAGGY insertions. (**A**) MAGGY can insert into MoTeR sequences in two orientations relative to the nearest chromosome end: i) sense and ii) antisense. The gray shaded area represents MoTeR2 sequence that has been disrupted by the MAGGY insertion. Hybridization probes were developed to detect each orientation: mS (sense; *Sac*II digest) and mA (antisense; *Afl*II digest). (**B**). Detection of new MAGGY insertions. Genomic DNAs from the single spore isolates were digested with *Sac*II or *Afl*II, size-fractionated by electrophoresis and probed sequentially with the telomere, mS and mA probes. The resulting phosphorimages are shown. Novel telomeric fragments that contain MAGGY sequences are highlighted. Left-hand panel: telomere probe; middle panel: mS probe; and right-hand panel: mA probe. With the telomere probe, note that certain TRFs have predictable size shifts between the *Afl*II and, *Sac*II digests, which represents the distance between the *Sac*II and *Afl*II sites in MAGGY. The orientation of each MAGGY insertion is easily determined based on whether the mS probe (dotted black ovals) or the mA probe (dotted white ovals) co-hybridized with the telomeric fragment.

To be sure of gaining a comprehensive insight into the various mechanisms underlying telomere rearrangements, we developed a hybridization-based method to pre-screen novel TRFs for MAGGY insertions. The genomic DNA samples were digested (separately) with *Afl*II and *Sac*II and then probed with internal MAGGY sequences, mA and mS (see Figure 4A). With these digestions and probes, MAGGY insertion in a MoTeR array is detected based on predictable band shifts when comparing the *Afl*II and *Sac*II digests: ∼1.2 kb for MAGGY insertions in the sense orientation, and ∼1.4 kb for antisense (Figure 4A). Orientation is then confirmed by the probe that hybridizes with the TRF (mA versus mS).

Among the nine single-spore cultures shown in the figure, the telomere probe identified at least 11 novel TRFs migrating at positions where the hybridization signals were clearly visible (see Figure 4B, left-hand panel, *Sac*II digest). Eight exhibited 1.2 or 1.4 kb size increases in the *Afl*II digest, consistent with the restriction site having been contributed via integration of MAGGY near the telomere. Insertion was confirmed by sequential probing with the mS and mA sequences: four of the novel TRFs hybridized with the mS probe (black dotted rings), indicating four, independent MAGGY insertions orientated away from the telomere (Figure 4A.i). The remaining four novel TRFs hybridized with mA (white dotted rings), consistent with three independent insertions oriented toward the telomere (Figure 4A.ii) (note that cultures 108 and 109 appear to be clones of one another).

Additional *de novo* MAGGY integrations were identified among MinION reads from fourth generation SS isolates. Isolate 2G4SS6–1 (hereafter, SS6–1) had a MAGGY insertion in a MoTeR1 element that formed part of an array in TEL-A (Supplementary Figure S10A), while 2G4SS15–11 (hereafter, SS15–11) had a MAGGY inserted in a MoTeR1 in TEL11. Interestingly, this latter element already contained an insertion of a copia-like retroelement, RETRO8 (Supplementary Figure S10B). Altogether, we identified twelve *de novo* MAGGY insertion events among 34 single-spore isolates that were screened and/or sequenced. With the exception of 3G1SS76, all insertion positions were 5′ of the MoTeR1 RT and MoTeR2 probe regions, which explained why MoTeR probes often failed to hybridize to the novel TRFs (data not shown).

### Break-induced expansion and contraction of MoTeR arrays

**Figure 5.**
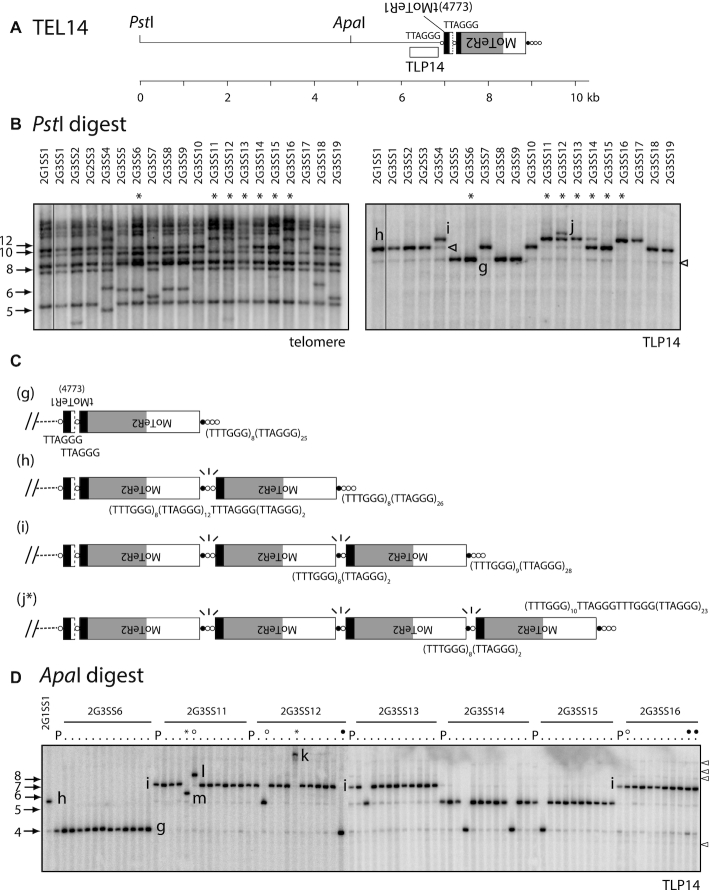
Recurrent rearrangements of TEL14 promoted by MoTeR-generated, interstitial telomeres. (**A**) Organization of the TRF containing TEL14. MoTeR2 segments are shaded to illustrate relevant portions: white = 5′ sequence shared with MoTeR1; light gray = sequence unique to MoTeR2; black = 3′ sequence shared with MoTeR1. The TLP14 probe was derived from chromosome-unique sequence immediately adjacent to the tMoTeR1 insertion. Telomeric circles are not drawn to scale so the ruler is approximate. (**B**) TLP14 identified multiple forms of the TRF in single-spore progeny. The left-hand panel shows a segment from a telomere-probed blot of single-spore cultures derived after two successive rounds of plant infection (54). The blot was stripped and re-hybridized with the TLP14 probe. The different TRF forms are labeled g, h, i and j. Cultures with the longer TRF variants were invariably heterokaryotic and contained some nuclei with shorter forms of the telomere (open arrowheads). Cultures used to generate the fourth generation single-spore families shown in C are labeled with asterisks. (**C**) Structural maps of TRFs g, h, i and j. Sequences of interstitial telomeres and telomere-like motifs are positioned below the relevant MoTeR-MoTeR junctions. Junctions without labels have the same sequence as the equivalent labeled junction above. Fragile MoTeR2-MoTeR2 junctions experiencing breaks are indicated with ‘\|/’ motifs. Structure j is denoted with an asterisk to indicate that it was inferred using MinION reads from another SS isolate (2G4SS4–20) and not by cloning. (**D**) TRF14 profiles for fourth-generation single-spore families (10–12 spores/generation) isolated from the third-generation SS cultures (see Supplementary Figure S1). DNA samples from the G4 cultures, their respective parents, and the original parent were digested with *Apa*I, fractionated by agarose gel electrophoresis, blotted to membranes and hybridized with the TLP14 probe (note: some minor fragments seen in B are missing because strains were re-cultured for DNA extraction). The image shows two separate blots that have been placed side-by-side. In the left-most lane of each blot is DNA from strain 2G1SS1. Then, for each family, the left-hand lane has DNA from the 3^rd^ generation parent (labeled ‘P’, with its identifier provided above the respective block) and DNA from the corresponding single-spore progeny to the right. TRF variants are labeled ‘g’ through ‘m.’ Lanes marked with open and closed circles, and asterisks are discussed in the text. Open arrowheads indicate additional telomere variants.

Shotgun cloning of TRFs from single-spore culture 1G2SS2 resulted in the recovery of an 8.7 kb fragment which contains TEL14. Restriction analysis and strategic sequencing revealed the telomere contains two MoTeR insertions, one a severely truncated MoTeR1 in the proximal position, and an intact MoTeR2 inserted distally (Figure 5A). All single-spore cultures, except for 2G3SS12, 13, 17 and 18, showed hybridization at the expected position (Figure 1, Supplementary Figure S2 and Figure 5B). However, when compared with the TEL5 bands, the varying signal strengths for TEL14 suggested that some cultures might possess co-migrating TRFs. Therefore, to monitor TEL14 in isolation, we used the telomere-linked probe, TLP14, to re-probe the telomere blot. In addition to expected signals from bands of 8.7 kb (band ‘g’) that corresponded to the cloned TRF, three additional signals were detected among the other SS isolates (bands ‘h’, ‘i’, ‘j’), with their sizes differing by increments of ∼1.7 kb—the length of MoTeR2 (Figure 5B). This pattern pointed to unusually high telomere instability, consistent with recurrent cycles of MoTeR2 gains and losses. This was confirmed through targeted cloning and sequencing of TEL14-h, -i, and -j variants from representative 3rd generation SS cultures, and by inspecting MinION reads for representative 4th generation SS cultures. Together, these sequences confirmed that the TRFs differed by incremental additions of MoTeR2 copies (Figure 5C and Supplementary Figure S11).

Work in other systems has shown that interstitial telomeres undergo frequent, spontaneous breakage (81–86) and, in fact, we and others have exploited this behavior as a facile way to engineer breaks in chromosomes (81,86). Because the shorter MoTeR arrays comprised precise truncations at MoTeR–MoTeR junctions, we reasoned that the interstitial repeats between elements promote breakage (Figure 2E), and then seed end-repair via telomerase-mediated telomere addition (‘telomere healing’). To test this prediction, we sub-cloned a number of TEL14 variants and sequenced the MoTeR-MoTeR junctions. In addition, we inspected interstitial telomere-containing MinION reads from three SS cultures. This confirmed that all junctions experiencing frequent breakage comprised long, interstitial, telomere-like/telomere tracts - the shortest examples being TTCGGG(TTTGGG)_8_(TTAGGG)_2_ (e.g. Figure 5C.i) and the longest, (TTTGGG)_5_TTAGGGTTTGGG(TTAGGG)_18_ (e.g., Figure 5C.h & Supplementary Figure S7.Ai). In contrast, the stable ‘g’ variant had just single TTAGGG motifs at the two MoTeR 3′ junctions. Critically, the MinION reads for TEL14 revealed variable (TTTGGG)n tract lengths between interstitial telomeres that occupied equivalent positions in TRFs with the same overall MoTeR array configuration (Supplementary Figure S11). This is most easily explained by replication slippage (Figure 2F)—a known mechanism underlying length variation in tandem repeat sequences (87). Given, the highly dynamic nature of TEL14, however, it is also possible that variation in tract length/composition arose through iterative cycles of break-induced replication (BIR).

Many single-spore isolates yielded multiple signals with the TLP14 probe, with most producing one strong signal and a number of fainter ones. Moreover, cultures with the longer ‘h’, ‘i’ and ‘j’ TRFs invariably showed weak hybridization at positions corresponding to one or more of the shorter forms (see Figure 5B, right-hand panel). This suggested that most fungal cultures were heterokaryotic for variant TEL14 forms despite recent purification via single-spore isolation. Furthermore, based on signal intensities, we estimated that ∼10 % of nuclei contained a truncated MoTeR array. To test this idea, we generated 4th generation SS families from 2G3SS isolates #6, 11, 12, 13, 14, 15 and 16 and examined their TRF14 profiles. For these experiments, the DNA samples were digested with *Apa*I which does not cut in MoTeR2 (see Figure 5A) and generates a smaller TRF that is better resolved during electrophoresis. As was predicted for heterokaryotic starting cultures (which, remember, were all originally derived from genetically purified SS cultures), five of the seven 4th generation spore families contained at least one SS culture where a shortened TEL14 TRF was now the dominant form. A good example is the culture 2G3SS12 which had ‘i’ as the major variant but also showed faint hybridization at the ‘g’ and ‘h’ positions (Figure 5D). In line with the relative intensities of the faint signals, one of the ten 4th generation SS cultures lacked hybridization to ‘i’ and contained variant ‘h’ as the predominant form instead (open circle), while a second culture showed exclusive hybridization to variant ‘g’ (closed circled). Interestingly, a third SS isolate from 2G3SS12 (asterisked) exhibited hybridization to a very high molecular weight band (‘k’) that was estimated to be >10 kb longer than fragment ‘j’ (Figure 5D). Here, it is important to note that the MoTeR1 sequence contains an *Apa*I site, so the large addition is unlikely to represent MoTeR1 acquisitions. The failure to detect shorter variants in the two exceptional families were likely due to the limited sampling of SS isolates.

Further evidence that the faint signals are due to heterokaryosis for variously truncated MoTeR arrays came from the observation that when ‘i’, ‘h’ and/or ‘g’ fragments co-occurred, their relative signal intensities varied. Clear examples of this are seen in the spore cultures from 2G3SS16, which showed strong hybridization to ‘i,’ clear signals in the ‘g’ position but little or no hybridization to ‘h’ (Figure 5D, ‘parental’ and open circle lanes). Likewise, cultures showing similar hybridization intensities for the major TRF often had varying signal strengths to the minor bands (e.g. the rightmost progeny from 2G3SS16—closed circles).

**Figure 6.**
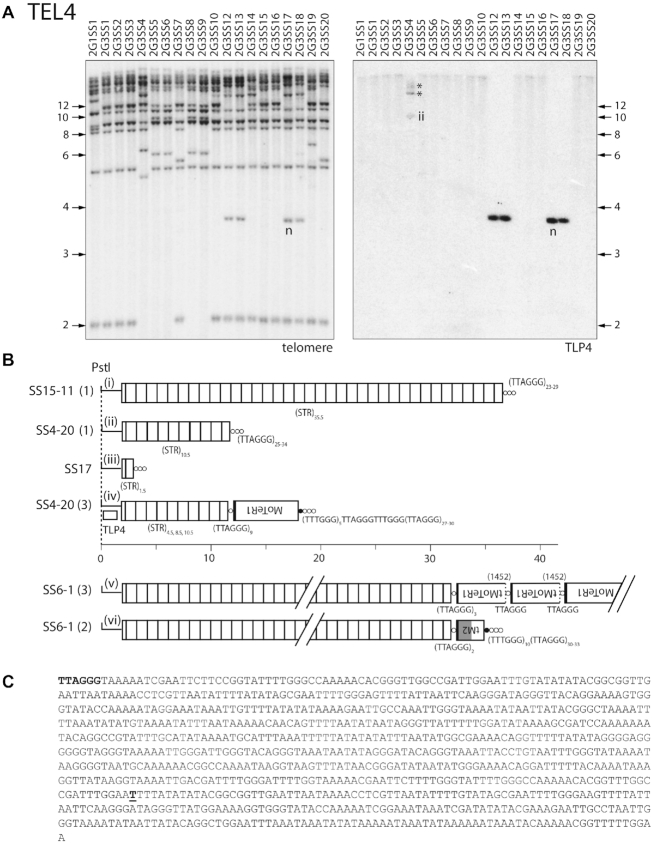
Subtelomeric repeat (STR) expansions/contractions, interstitial telomere breaks and MoTeR acquisitions at TEL4. (**A**) Southern hybridization analysis of SS progeny showing novel TRF-n (left-hand panel) and monitoring instability at the corresponding telomere (TEL4) using TLP-n (right-hand panel). Asterisks mark bands corresponding to different length variants shown in B.iv. (**B**) TRF structures elucidated via MinION sequencing of SS4–20, 6–1 and 15–11 and Sanger sequencing of TRF-n. MoTeR arrays are annotated according to the standard schema. Individual units of a tandem repeat array found in the subterminal region of TEL4 are represented as open boxes. MinION sequencing identified distinct TRF structural variants in SS4–20 and SS6–1, while SS15–11 contained a single TRF form. The TRF structure for SS17 was inferred from Sanger sequence data. All structures shown as fully contiguous entities were inferred from individual MinION reads that spanned the entire distance from the chromosome unique sequence to the telomere. Inferred rearrangement mechanisms: i → ii → iii: intrachromatid recombination, unequal sister chromatid exchange or gene conversion; ii → v) combination of MoTeR acquisition via ectopic recombination with TEL8 and array shortening via intrachromatid recombination, unequal sister chromatid exchange or gene conversion. (**C**) Nucleotide sequence of an individual tandem repeat unit. The single TTAGGG repeat unit is shown in bold and the T nucleotide at which the actual telomere starts in all of the SS cultures is marked with bold, underlined text.

Telomeres 6, 12, A, B, and D also underwent rearrangements that also appeared to have been simple capping events following interstitial telomere break (Supplementary Figures S7, S10A, S12 and S13). On the other hand, telomeres 2, 3, 4, 7, 12, B, as well as variants of TEL14 identified among MinION reads, exhibited alterations resembling simple ‘break->cappings’ but, on closer inspection, duplications of specific variant telomere motifs pointed to the occurrence of more complex repair mechanisms involving invasion of sister chromatids/other telomeres and/or D-loop formation (Figure 6, Supplementary Figures S4, S7, S11, S13, S15).

### ‘Contraction/expansion’ of subterminal tandem repeats

Single-spore cultures 2G3SS12, 2G3SS13, 2G3SS17 and 2G3SS18 exhibited a novel TRF at a molecular size corresponding to ∼3.7 kb (band ‘n’, Supplementary Figure S2 & Figure 6A). The TRF in the 2G3SS17 culture was cloned and subjected to end-sequencing, which revealed a simple telomere at one end of the insert (Figure 6B.iii, SS17). Comparison with the B71 reference genome identified this as TEL4. To identify the corresponding TRF in the other SS isolates, the blot was stripped and re-probed with a TEL4-linked probe (Figure 6A). As expected, TLP4 hybridized strongly to the 3.7 kb fragments but produced very faint, high molecular weight bands with smeared tails in the other lanes (Figure 6A, right-hand panel), indicating possible degradation of high molecular weight fragments. Next, we digested agarose-embedded, intact chromosome preparations (15,88) with *Pst*I, and fractionated the products using contour-clamped homogeneous electric field (CHEF) electrophoresis, and hybridized blots sequentially with TLP4, telomere and MoTeR probes. TLP4 hybridized to large fragments that were more than 45 kb in length and whose sizes appeared to vary slightly among different chromosome ends (Supplementary Figure S16A). Additionally, cultures 2G1SS1 and 2G3SS1 exhibited a number of faint signals to shorter fragments (arrowheads) signaling the existence of rearranged TRFs within these cultures. Interestingly, in cultures 2G1SS1, 2G3SS14 and 2G3SS20, the TLP4-hybridizing fragments did not hybridize with the telomere and MoTeR probes and are, therefore, not telomeric.

Inspection of MinION reads for the fourth generation SS cultures SS4–20, SS6–1 and SS15–11 indicated that the TEL4 *Pst*I fragments were telomeric in these strains, and contained many copies of a ∼0.9 kb subtelomeric tandem repeat (STR) between the *Pst*I site and the telomere (Figure 6B and C). For SS15–11, a complete *Pst*I TRF was captured in a single MinION read, which contained 35.5 STR copies and terminated in a telomere (Figure 6B.i). This was in striking contrast to the 3.7 kb TEL4 fragment cloned from SS17 which contained just 1.5 repeats (Figure 6B.iii). A Southern blot of *Pst*I-digested DNA from the SS15–11 MinION DNA samples suggested that TRF4 was mostly stable in this particular strain, although a faint signal was observed at a molecular size of ∼17 kb (Supplementary Figure S16B).

Four MinION reads from SS4–20 spanned entire STR arrays from the unique TLP-4 region to the telomere and, in doing so, revealed wide variation in STR lengths (4.5 to 10.5 repeat units, Figure 6B, variants ii and iv), which was also evident in a *Pst*I blot of the MinION DNA sample (Supplementary Figure S16B). For SS6–1, it was not possible to assess length variation with the MinION data because no reads spanned an entire STR array. However, the presence of an indistinct smear in the Southern blot of the SS6–1 DNA used for MinION sequencing indicates frequent rearrangement in this strain too (Supplementary Figure S16B). In addition to STR length differences, SS4–20 also varied in presence/absence of a MoTeR1 insertion in the telomere (Figure 6B.ii versus iv), whereas all five of the telomere-containing reads in SS6–1 had MoTeR insertions, although the composition and array lengths varied (Figure 6B.v and vi). Critically, all of the telomere-containing reads exhibited identical boundaries between the telomere repeats and adjacent STR. From this, we conclude that most TRF4 rearrangements were unrelated to the telomere *per se* and, instead, involved contractions/expansions of the STR array—likely via unequal intra-chromatid and/or unequal sister chromatid exchanges (Figure 2G).

Additional examples of subterminal repeat expansion and contraction without alterations at the chromosome terminus were observed in the TEL8 subtelomere (Supplementary Figure S6A) and in a MoTeR array at TEL10 (Supplementary Figure S14).

### Simple terminal truncations

**Figure 7.**
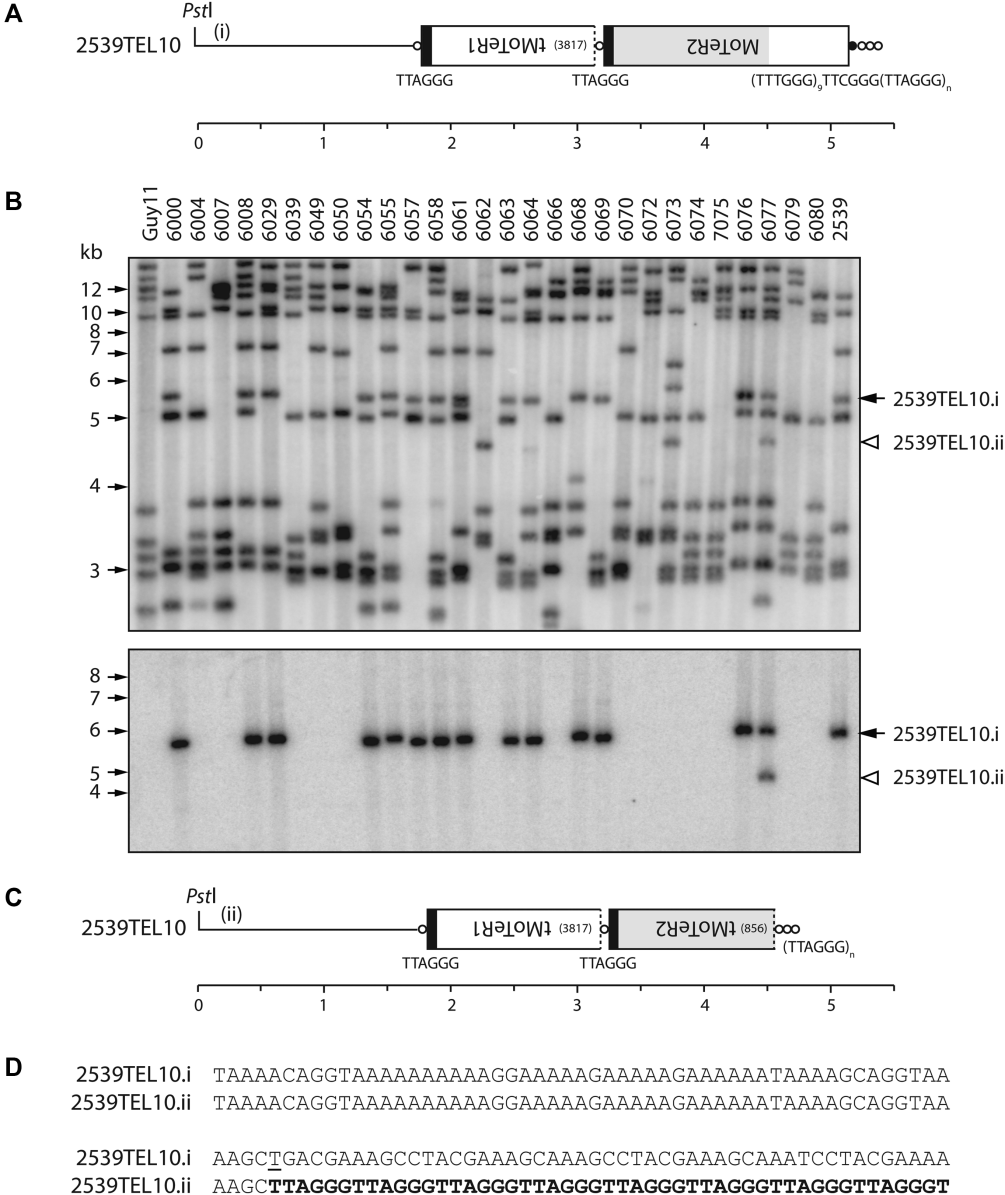
Simple terminal truncations. (**A**) Telomeric *Pst*I fragment containing 2539TEL10 showing MoTeR sequences embedded in the telomere. (**B**) TRF segregation among progeny of a cross between 2539 (MoTeR^+^) and Guy11 (MoTeR^−^). The top panel shows the phosphorimage of a Southern hybridization analysis of *Pst*I-digested DNAs probed with telomere repeats. Parental strain DNAs are loaded in the outermost lanes, with progeny DNAs in between. 2539TEL10.i is marked with a black arrowhead. A white arrowhead marks a novel TRF that corresponds to a TEL10 variant (ii). Bottom panel: the membrane used in A was stripped and re-hybridized with a MoTeR2 probe. (**C**) Organization of 2539TEL10.ii showing 5′-truncation of the terminal MoTeR2. (**D**) 106 bp of sequence surrounding the site of *de novo* telomere addition at the MoTeR2 truncation boundary. Telomere repeats are highlighted with bold text. A ‘T’ nucleotide that seeded the *de novo* telomere addition is underlined. Inferred rearrangement mechanism: i → ii) MoTeR truncation, *de novo* telomere formation.

Another type of alteration in MoTeR-containing telomeres was identified in a separate study involving analysis of progeny from a cross between a MoTeR-lacking strain of *M. oryzae*, Guy11, and strain 2539, which has three MoTeR-containing telomeres. 2539 has a single, intact MoTeR2 copy inserted distal to a truncated MoTeR1 in TEL10 (Figure 7A). Southern hybridization analysis using MoTeR2 to probe *Pst*I-digested DNAs from 52 progeny of a cross between 2539 and Guy11 revealed a single alteration among 25 progeny that inherited this telomere (representative isolates are shown Figure 7B). Progeny 6077 contained two MoTeR2-hybridizing bands, with the second one hybridizing at a slightly weaker intensity. Cloning of the new TRF from strain 6077 revealed that it is identical in structure to TRF10, except that the MoTeR2 had suffered a terminal truncation (Figure 2H) that eliminated 856 nucleotides from the 5′ end (Figure 7C and D), with the new telomere having been extended from an in-frame T nucleotide which presumably acted as a telomeric seed. Inspection of 22 pre-existing 5′ truncated MoTeRs showed that 17 of 23 had in-frame seed sequences (1–7 nucleotides) at their junctions with the telomere repeats, and in five of the six exceptions there was a C at the terminal position (Supplementary Table S4).

Two additional, newly-arisen, terminal truncations were detected among the LpKY97 single spores - one, involving TEL14, occurred in one of the 4th generation SS cultures (SS11–4) (Figure 5D) and another was at TEL2 (Supplementary Figure S13A).

### Tandem amplification via D-loop formation

All MinION reads containing TEL3 had an intact MoTeR1 inserted at a proximal position in the telomere (Supplementary Figure S15A). In SS4–20, there were between one and five distal copies of MoTeR2. Structurally, this organization resembled that of TEL14 (Supplementary Figure S11). However, unlike TEL14, and virtually all other multi-MoTeR arrays, the lengths of interstitial telomeres and variant repeats separating element copies were identical, making it unlikely that array extensions occur via sequential transposition, or through recurrent interstitial telomere breakage. Instead, it appears that they arose through sequential cycles of array extension, following D-loop formation by the broken end (Figure 2I and Supplementary Figure S15B). To explain the precise duplications of interstitial telomeres, each amplification cycle would have to be initiated by free ends comprising internal sequences common to MoTeR1 and MoTeR2 because naked telomere repeats would have the capacity to invade in random registers. Alternatively, identical repeats could be generated through a rolling circle mechanism, as has been demonstrated for TERT-minus strains of *Kluyveromyces lactis* (89,90). Here, the first break—D-loop formation—extension cycle would generate an extrachromosomal circle that could serve as a template for potentially ‘infinite’ extension of free ends that invade complementary sequences within the circle (Supplementary Figure S15C) The structures shown in Supplementary Figure S15A would represent products arising from increasing numbers of replication fork circumnavigations, followed by telomere capping of the final products.

A MoTeR1/2 array in TEL-D has a structure consistent with a historical ‘break, D-loop formation’ event. The array contains 18 different MoTeR copies and exhibits a repeating tMoTeR1–tMoTeR2–tMoTeR2 pattern, in which the MoTeR truncation positions, as well as the interstitial telomere compositions at specific tMoTeR–tMoTeR junctions, are identical throughout the array (Supplementary Figure S5). The one exception involves variation in the repeat pattern for MoTeR2 in the proximal portion of the array, which likely arose through an intra-/unequal sister chromatid exchange.

### Stable telomeres

Most TRFs showed rearrangements in a number of single spore (SS) cultures, with the notable exception of TRF5 (containing TEL5) (Figure 1). TRF5 was relatively stable and exhibited rearrangement in only two out of more than 250 SS isolates that were analyzed (Figure 1, Supplementary Figure S2; and data not shown). In addition to TEL5, TELs 9, 11 and 13 were also relatively stable (Supplementary Figures S6B & S10B), as evidenced by inspection of Southern blot data and MinION reads. TEL9 was missing in only four out of 143 SS isolates (Figure 1, 1G3SS1 and 1G3SS4; Supplementary Figure S2). For TEL11, no rearrangements that were directly attributable to MoTeR-driven processes - but one of 37 MinION reads identified a MAGGY insertion in a MoTeR array (Supplementary Figure S10B). The general organization of these ‘stable’ chromosome ends comprised a telomere with a single intact, or truncated, MoTeR1; the interstitial telomere tract contained no more than two repeats; and the canonical telomeres were attached to truncated MoTeR sequences, or TTTGGG tracts of various lengths (Supplementary Figure S6B). Together these data suggest that the TTCGGG(TTTGGG)_*n*_ tracts at the 5′ ends of intact MoTeR insertions do not compromise telomere integrity by themselves, and that two consecutive TTAGGG repeats are insufficient to promote breaks. Although the general structure of the ‘stable’ telomeres remained largely unaltered, varying numbers of TTTGGG repeats were sometimes found at the MoTeR 5′ termini (see Figure 9B). These changes are not easily explained by recurrent cycles of break/repair as this ought to result in collateral alteration of the adjacent, and rather unusual, TTAGGGTTTGGG motif. Instead, we propose that alterations in short tandem repeat tracts occur via replication slippage (87).

### The LpKY97 genome has a history of MoTeR-associated chromosome rearrangements

**Figure 8.**
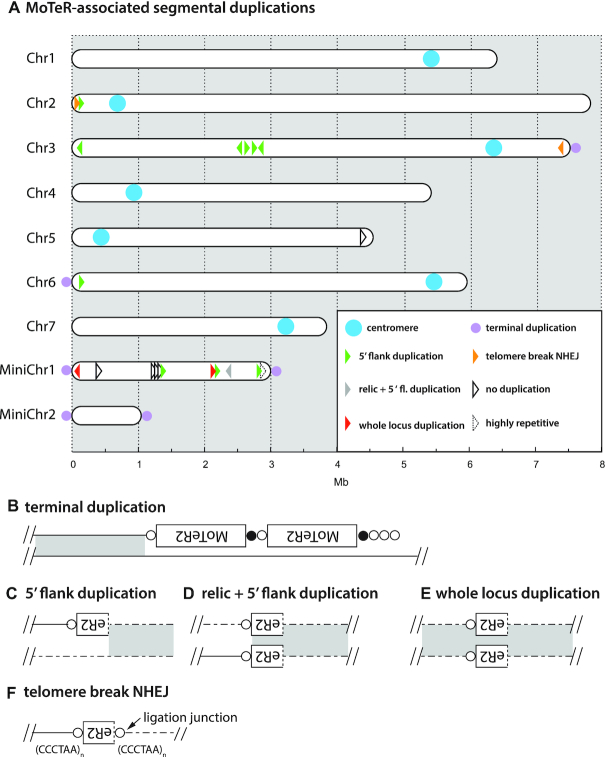
MoTeR-associated, segmental duplications in chromosomes of LpKY97. (**A**) Locations of MoTeR relics in the LpKY97 genome. Each element is represented with an arrowhead representing the relic's orientation (5′ to 3′). The colors show the nature of the duplication as shown in the legend. The types of duplications observed were: (**B**) sequences at chromosome ends, immediately adjacent to terminal MoTeR arrays—indicated with lilac dots on the respective chromosome ends; (**C**) 5′-flank duplications beginning at, and extending out from, a relic's 5′ truncation boundary, (**D**) duplication of the relic and the 5′-flanking sequence; (**E**), duplication of the entire locus encompassing a relic. Also shown are examples of putative NHEJ involving broken interstitial telomeres (**F**). Relics embedded in single copy DNA are shown in black. One insertion was flanked by a highly repeated transposon sequence.

**Figure 9.**
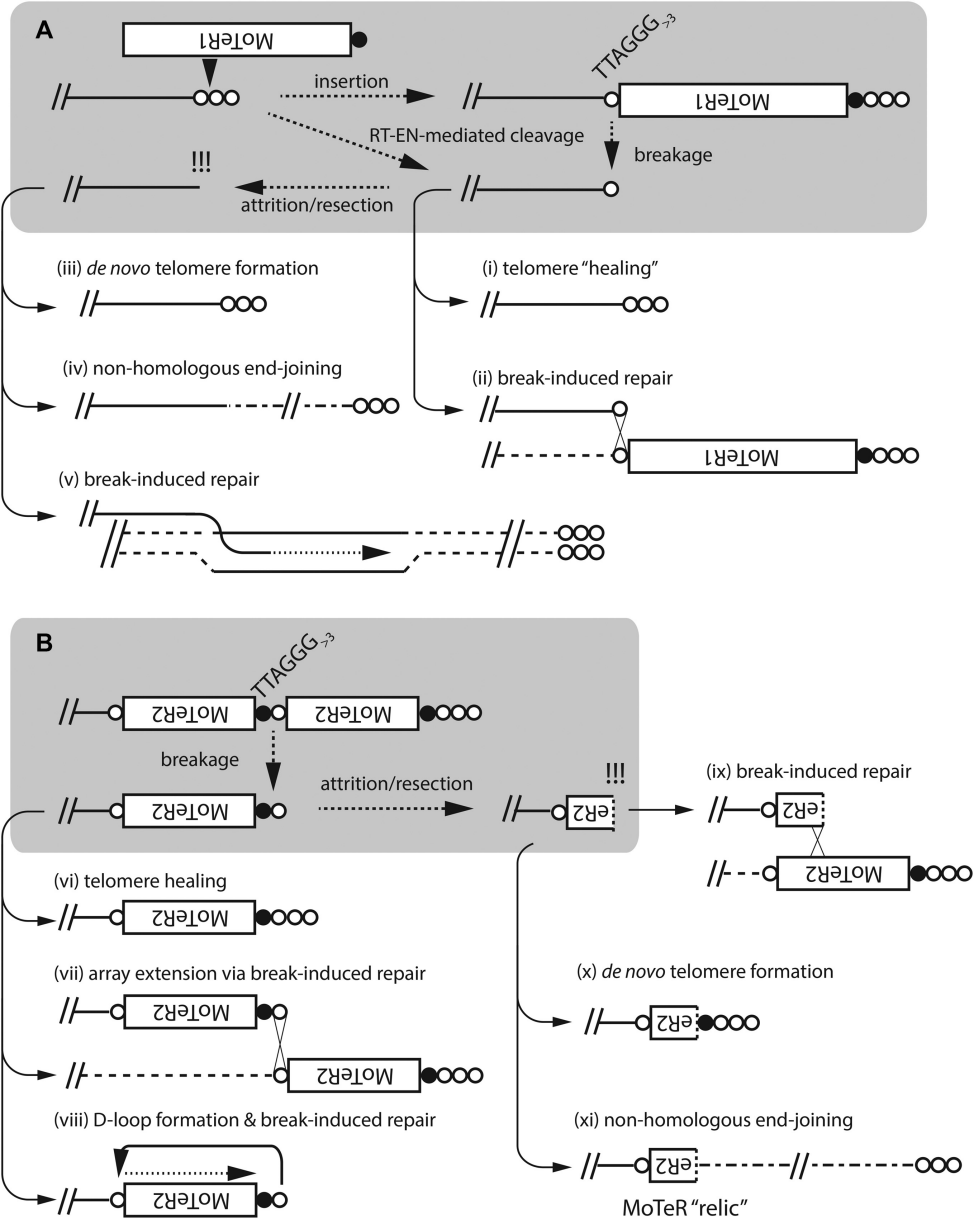
Schematic showing how MoTeR insertions can promote re-organization of the genome interior. (**A**) *End-deprotection*. If a MoTeR inserts in a plain telomere at a position more than three TTAGGG repeats distal to the telomere boundary, this generates a fragile site. Subsequent breakage would result in the creation of a de-protected end. Alternatively, end de-protection could occur directly via telomere cleavage by the site-specific endonuclease in the MoTeR reverse transcriptase, without a subsequent reverse transcription step. If repair is initiated before the remaining telomere repeats are lost through replicative attrition, or enzymatic resection, the ends could be repaired by telomere ‘healing’ (i), or by ectopic invasion of homologous sequences on a sister chromatid or another chromosome end (ii). In the case shown, the donor telomere possesses a MoTeR insertion, resulting in MoTeR acquisition (ii). Delayed repair could result in loss of subterminal sequences through attrition/resection, resulting in the generation of a ‘naked,’ recombinogenic end and, possibly, extensive terminal sequence loss. These naked ends can be repaired by *de novo* telomere formation (iii), non homologous end-joining (NHEJ) (iv), or break-induced replication (BIR) - if the free end has homology to another chromosomal sequence (v). (**B**) *MoTeR array ‘activation.’* Breaks at interstitial telomeres constituting MoTeR-MoTeR junctions can be repaired via healing (vi); BIR using other MoTeR arrays as templates, resulting in array extensions (or contractions) (vii). Alternatively, the free end can invade the same DNA strand, forming a D-loop, with the end being extended by BIR (viii). Finally, attrition/resection of the new terminus will produce a free end comprising internal MoTeR sequences (a truncated MoTeR). This can be repaired via *de novo* telomere addition (ix), BIR (x), or NHEJ (xi). Note that NHEJ will result in the internalization of the truncated version of a formerly telomeric MoTeR. We refer to these internalized elements as MoTeR ‘relics.’

Many *M. oryzae* strains lack full-length MoTeR copies but, when intact elements are present, they are only found embedded in telomeres (54; this study). On the other hand, all strains possess short relics of MoTeR sequences residing at internal chromosome locations. These relics comprise MoTeR 3′ termini attached to a short stretch of telomere sequence (Supplementary Table S5) and, as such, they serve as tags for sequences that were once telomeric (54,55). To explore possible mechanisms by which MoTeR relics become internalized, and thereby alter genome structure, we examined the chromosomal neighborhoods of the 19 internal MoTeR relics that present in the LpKY97 genome (Figure 8A, Supplementary Table S5). Twelve loci had 5′ flanking sequences that were duplicated, with the duplications starting at the MoTeR 5′ boundary and extended into neighboring DNA (see Figure 8B). Nine of these structures are consistent with a scenario in which telomeric MoTeRs were resected and underwent non-homologous end-joining (NHEJ) with internal sequences that became duplicated in the process (Figure 8C). The three remaining relics formed a tandem repeat in the middle of mini-chromosome 1, where they were interspersed with non-MoTeR DNA (Figure 8A) - a structure that was likely generated through iterative cycles of BIR via D-loop formation (or copying from an extrachromosomal circle), when the 3′ MoTeR copy was still positioned at a chromosome end, or the end of a broken molecule. All MoTeR relics have telomere repeats (or at least a partial repeat) at their 3′ termini. Unexpectedly, the LpKY97 genome harbored two relics that also had TTAGGG tracts at their 5′ truncation boundaries (see Figure 8F). This arrangement is consistent with a scenario in which breaks occurred in interstitial telomeres at 5′-truncated MoTeR borders and underwent NHEJ, resulting in the internalization of the relic (see Figure 9). One relic resides in the middle of a segmental duplication (Figure 8D) and was likely a passive passenger in an unrelated rearrangement. Finally, five relics were flanked on both sides by single copy sequences, signaling their possible immigration to internal chromosome locations via translocation. Alternatively, it may be that duplications were originally involved but the original template copies were subsequently lost. In this regard, it is perhaps significant that strain LpKY97 comes from an *M. oryzae* population with a history of genome shuffling through admixture, which has caused a number of segmental duplications to be purged from some population members (M. Farman, unpublished data).

## CONCLUSIONS

With this study, we gained a comprehensive insight into the molecular basis for frequent TRF rearrangements associated with the presence of MoTeRs inserted in the telomeres of *M. oryzae*. Altogether, 109 telomere/subterminal rearrangements were identified and characterized at the molecular level (Supplementary Table S6). Previously, we showed that rearrangements were largely restricted to TRFs in which the telomeres contained MoTeR insertions - TRFs lacking MoTeRs were generally quite stable, with the lone exception of the rDNA telomere (54). While our current data are in overall agreement with these earlier findings, we discovered that MoTeR presence *per se* is neither necessary nor sufficient to promote frequent terminal alterations.

Three chromosome ends exhibited frequent rearrangement in the absence of MoTeR sequences. Consistent with our previous observations, 27 independent rDNA telomere rearrangements were detected through the characterization of altered TRFs and analysis of MinION reads. Most of these changes occurred at ends lacking MoTeR insertions. The 28S rRNA gene array is telomeric in several organisms ranging from fungi to plants (18,19,68,76) and, in fungi, terminal truncations are frequently detected ((18,54,82,91) and this study). Recently, it has been shown that silent rRNA copies are replicated in mid to late S-phase and have a compact heterochromatin structure (92), which possibly promotes replication stress and subsequent rDNA breakage (93). It seems reasonable to suppose that *M. oryzae* experiences even higher replication stress in the telomere-adjacent rDNA copies due to a heterochromatin structure resulting from a telomere position effect, and the resulting breaks would then be repaired via *de novo* telomere formation.

We also demonstrated MoTeR-independent instability in a newly-discovered tandem repeat present in subtelomeres 4 and 8. Contrary to the rDNA, however, this STR, which comprises a much shorter repeat unit, and whose function (if any) is unknown, showed TRF length variation in the absence of terminal truncations. This implicates a novel mechanism that does not appear to involve a compromised telomere. In yeast, expansion and contractions of the rDNA repeat are initiated following breakage at replication fork barriers (94), and the resulting breaks are usually repaired via gene conversion, although unequal sister chromatid exchanges are also seen (95). The high frequency of STR contractions/expansions in *M. oryzae* implies the existence of a strong replication fork block in these sequences. Why STR breaks should be repaired via gene conversion/unequal crossover, as opposed to *de novo* telomere addition, as is the norm for the rDNA, is unclear. However, in humans, there is evidence that the telomere-associated Bloom helicase also interacts with rDNA sequences (96), which possibly indicates a role for related helicases specifically in the healing of rDNA breaks.

Just as MoTeR presence is not necessary for instability to occur, their presence also does not guarantee it. One MoTeR-containing telomere in LpKY97 showed no evidence of alterations (TEL 13), while three others (TELs 5, 9 and 11) showed low frequency changes. This shows that the MoTeR sequences *per se* do not compromise the stability or the integrity of the chromosome termini. This is key because both elements contain several unusual sequence motifs, including highly T-rich tracts, blocks of tandem repeats and, most significantly, telomere-like repeats at their 5′ ends (54). Initially, we suspected that these TTTGGG repeats might destabilize the protective telosome, causing the telomere to become compromised. However, the rare alterations in TELs 5, 9, 11 and 13 indicates that telomere attrition is not a significant factor in driving alterations.

Instead, the key property that distinguished a stable, MoTeR-containing telomere from an unstable one was the absence of an extended interstitial telomere tract between elements. Stable interstitial telomeres had a maximal tract length of (TTAGGG)_2_ (minimal: AGGG), while most of the highly unstable telomeres had much longer inter-MoTeR tracts, with the longest comprising 18 TTAGGG/TTTAGGG repeats attached to the variant repeats found at the MoTeR 5′ ends (consensus: TTCGGG[TTTGGG]_8_TTAGGGTTTGGG). From this we conclude that interstitial telomeres with three or more TTAGGGs are sufficient to promote replication stalling and subsequent breakage. Additional, albeit indirect, evidence that three repeat units are sufficient to promote breaks comes from the observation that (TTAGGG)_3_ tracts (or permutations thereof) are entirely absent from internal locations in the majority of *M. oryzae* genomes (data not shown). The lone exceptions are *M. oryzae* strains from foxtail grasses (*Setaria* spp.) which often have numerous long, interstitial telomeres in their subtelomere regions, which likely arise from bouts of spontaneous failure in telomere maintenance (A. Yackzan, M. Rahnama and M. Farman, unpublished data). Such strains exhibit extremely frequent TRF alterations, despite the fact that they lack MoTeRs (55).

MoTeR1 codes for a reverse transcriptase with a restriction endonuclease-like (REL-ENDO) domain, which suggests that it is a site-specific transposon that targets telomeres (54). Several of the rearrangements described here involved MoTeR acquisition by a plain telomere, or the addition of new MoTeR copies at the end of an existing array. While both types of occurrences can be explained via transposition, we cannot rule out the possibility that new MoTeRs were copied from other chromosome ends, or from sister chromatids, via BIR (see below). Normally, we would not expect ‘plain’ telomeres to acquire *de novo* MoTeR insertions by BIR because, with the exception of the rDNA, such telomeres are very stable, indicating that recombinogenic end formation through de-protection rarely occurs. As an example, when surveying for telomere alterations in *M. oryzae* rice pathogens that lack MoTeRs, only 14 novel TRFs were detected among more than 1,200 fragments surveyed (41,54,55), and most, if not all, of these were almost certainly due to rDNA truncations. In a similar vein, we do not expect MoTeR-containing telomeres with short interstitial TTAGGG/TTTGGG tracts (e.g. TEL5 & TEL-C) to experience spontaneous breakage. For these reasons, we suspect that MoTeR acquisitions by otherwise stable telomeres possibly originated via transposition. In this regard, we hypothesize that the MoTeR RT protein has the ability to access and cleave telomere repeats that would otherwise be highly protected - an ability that might be further enhanced through interaction with the (TTTGGG)_*n*_ motifs at MoTeR 5′ ends. Ultimately, however, formal proof that specific rearrangements are due to MoTeR transposition, and an estimation of the frequency of such events, will require the use of a definitive retrotransposition assay (97). Finally, if the MoTeR RT or, more specifically, the predicted endonuclease does have access to an otherwise protected telomere (as is implied by the MoTeRs' residence therein), then there is also the possibility that it could promote recombination-mediated re-arrangements by generating free DNA ends through telomere cleavage without attendant reverse transcription.

While we were unable to provide conclusive demonstration of *de novo* MoTeR transposition events, several TRF alterations were attributable to new insertions of the MAGGY retrotransposon (98) into MoTeR arrays. The MAGGY reverse transcriptase contains a chromodomain which targets insertions to heterochromatic regions (99). This suggests that MoTeR arrays might inherit a heterochromatic state from the telomeric regions into which they insert. Given that MoTeR sequences were such attractive MAGGY targets, it is perhaps surprising that we never identified MAGGYs in any of the many MoTeR copies that we characterized previously (54) (and data not shown). Similarly, other *M. oryzae* strains had remarkably stable telomeres even when their genomes contained multiple MAGGY copies (unpublished data). It appears that the element may have been uniquely activated in the particular LpKY97 cultures under study. MAGGY is known to be more active under stress conditions (100) so it is possible that the particular culture of LpKY97 under study suffered an unintended stress.

Transposons can promote genome evolution and organismal adaptation through a variety of mechanisms. In *M. oryzae*, simple insertions of transposons into genes and gene promoters have played key roles in the fungus' ability to infect new hosts. As an example, the recently emerged wheat blast disease is caused by highly virulent fungal lineages whose evolution was facilitated by three separate inactivations of *Pwt3* - an avirulence gene whose protein product normally triggers resistance in plants carrying the *Rwt3* resistance gene (8). Likewise, transposon insertions in two different avirulence genes led to the emergence of strains that gained virulence to rice cultivars that were formerly resistant (12,101 ). In addition to simple insertional inactivation/activation of genes, transposons can also induce rearrangements with broader impacts on genome content and architecture. Aberrant transposition events can produce a variety of major chromosomal rearrangements, including translocations, deletions, duplications and inversions (71). The same types of rearrangements can also occur long after transposition has occurred, when normal cellular recombination and repair processes act ectopically on dispersed repeats that are generated through transposition activity (64,102–104). Again, in *M. oryzae*, various deletions that resulted from recombination between transposon copies in and around the *AVR1-CO39* gene were responsible for defeating the *Pi-CO39* resistance gene which prevents infection of rice by *M. oryzae* from other grass species (13). Thus, transposon-mediated genomic alterations also appear to have been primary drivers of *M. oryzae'*s ability to colonize rice (68 ). In fungi, the repeated sequences that result from transposition activity can also drive gene evolution via a mutagenic process known as repeat-induced point mutation (RIP). RIP operates during sexual development and causes mutational sweeps in multi-copy sequences (105). While the primary role of RIP is believed to be transposon inactivation, it also operates on genes in segmental duplications (106), and can leak into neighboring single-copy sequences, thereby accelerating their functional diversification (107,108 ). Finally, transposons can affect the genome's adaptive capacity by determining the heterochromatin landscape (109) and by promoting the epigenetic regulation of neighboring genes (110). Together, the potential for transposons to generate variation in gene content and expression, may explain why several *M. oryzae* avirulence genes, that are so crucial in determining host infection capability, have transposon insertions in their promoter regions (111–113).

In the present study, we identify an entirely new mechanism by which transposons can drive genome evolution. When a MoTeR inserts into a telomere, it inevitably causes a portion of the target site to become internalized, producing an interstitial telomere (Figure 9A). Critically, it appears that interstitial telomeres with as few as three TTAGGG repeats (or just two, when attached to variant repeats at MoTeR 5′ ends) can promote breakage, with the frequencies of such events reaching ∼10% or more (e.g. Figure 5). In yeast, it has been shown that the repair of such breaks can cause genome rearrangements such as deletions, duplications, inversions, and translocations; and can also generate acentric minichromosomes (114). For the MoTeR-associated breaks in *M. oryzae*, collateral genome effects will likely depend on the nature of the break and the mechanism of its repair: If the proximal side of a broken telomere still contains a vestige of TTAGGG repeats, the end can probably be restored through telomerase action (Figure 9A.i), or by BIR using another MoTeR array as template (Figure 9A.ii). From the TEL14 signal intensities in Figure 5D, which show very clearly that arrays shorten much more often than they lengthen, we conclude that most interstitial telomere breaks are simply healed. Nevertheless, the occasional appearance of longer arrays in some cases, points to the operation of other repair processes or, possibly, MoTeR transposition.

Break-induced replication (BIR) was evidently involved in some instances of MoTeR array elongation because the newly acquired elements possessed features characteristic of specific MoTeR copies in other telomeres. Examples included MoTeRs that were truncated at specific nucleotide positions, or copies flanked by variant repeat tracts with unusual (TTTGGG)_*n*_TTAGGG configurations. The operation of BIR was not all that surprising because broken interstitial telomeres should be structurally equivalent to the shortened telomeres that result from experimental deletion of the telomerase (TERT) and, in yeasts and humans, BIR is the default repair pathway for telomeres thus compromised (115–117). Moreover, *M. oryzae* TERT knockout strains preferentially repair their telomeres via BIR, wherein critically shortened telomeric 3′ ends invade their own subtelomeres at very short TTAGGG motifs to form a D-loop. The telomere ‘tail’ then primes replication which extends to the chromosome end. Tandem amplification of the newly synthesized sequence then others, either by re-iteration of this or process, or through invasion/replication on extrachromosomal circles generated in an earlier D-loop formation/extension event (89,90) (Peppers, Rahnama and Farman, unpublished data). For the interstitial telomere breaks observed here, however, it is easy to imagine that once BIR extends to the telomere on the invaded, template strand, the new end thus acquired should be easily repairable through normal telomerase action.

If breaks occur in a very short interstitial telomere, or at the proximal end of an extended one, this would produce a very short telomere vestige that may fail to engage telomerase, causing the adjacent sequences to be lost through replicative attrition, or enzymatic resection (Figure 9A). The fate of such ends, and the possibility of wider chromosomal alterations, likely depends on the nature of the terminal sequences. If breaks occur at the boundary of an interstitial telomere and chromosome-unique sequence, attrition/resection will probably result in loss of subterminal sequence and the generation of a recombinogenic end with a number of potential pathways to repair. telomere seed (minimally, T or A) may allow for end-repair via *de novo* telomere formation (Figure 9.iii) (118). Alternatively, if the ends occur in single copy DNA, they may participate in NHEJ (Figure 9.iv) (119); or, if they comprise repeated sequences, they potentially could invade a homologous locus and undergo BIR (v). Both of the latter events have the potential to result in the capture and duplication of internal sequence at the chromosome end.

Most of the breaks characterized here occurred in fragile sites that constituted MoTeR-MoTeR junctions (Figure 9B). Intuitively, these seem less likely to produce major chromosomal alterations. Not only should the proximal MoTeR sequences act as buffers against loss/exposure of the chromosome-unique sequences, but they should also provide abundant opportunities for repair: First, the exposed telomere repeats can be simply healed (Figure 9.vi) or, second, they could initiate BIR by invading interstitial telomeres between other MoTeRs (Figure 9.vii). Finally, there is the potential for D-loop formation through the invasion of proximal interstitial telomeres on the same DNA strand. In this case, the resulting BIR would lead to a precise duplication of the proximal element (Figure 9.viii). Should the telomere repeats be lost through attrition/resection, the MoTeR sequences at the ends should still be eligible for BIR (Figure 9.ix), or *de novo* telomere formation (Figure 9.x). Given the multiple routes by which MoTeR-terminated free ends could be repaired, it might seem unlikely that they would undergo NHEJ (Figure 9.xi). However, the presence of multiple MoTeR relics at internal chromosome locations (Figure 8A) indicates that such occurrences are, in fact, quite common.

MoTeR-induced breaks that undergo NHEJ, or utilize internal loci as BIR templates, have the potential to cause internal sequences to be duplicated at, or translocated to, chromosome ends. In a prior study, we were able to pin-point the boundary of one such duplication to a transposon present in both the terminal and interior locations, directly implicating BIR as the driving mechanism (18). Unfortunately, we were unable to characterize the boundary of the duplication that gave rise to the novel TRF-C, but it seems reasonable to suppose that a MoTeR-induced break might have been the initiating event. Evidence supporting direct MoTeR involvement in the creation of segmental duplications in the LpKY97 genome came from 12 internal relics that defined duplication boundaries (Figure 8) and, therefore, serve as historical markers of terminally-directed duplication events.

Segmental duplications play important roles in gene and genome evolution, by providing raw materials for functional diversification through the creation and expansion of gene families (120,121). By generating fragile sites at chromosome ends, MoTeRs not only promote repair-driven duplication/translocation events, but they direct these processes to the chromosome regions with the greatest evolutionary potential. Thus, one can imagine a dynamic in which genes are duplicated in the subterminal regions where they experience accelerated evolution and, as result, have an enhanced potential for neo-functionalization. Moreover, newly-evolved genes with useful functions then have a means of being recruited back to the more stable genome interior, via subsequent terminalization events. Thus, the impressive telomere dynamicism generated through MoTeR invasion of telomeres can be leveraged for maximal evolutionary benefit.

## DATA AVAILABILITY

The merged, SNP-corrected, MinION genome assembly for LpKY97 has been deposited at NCBI under the BioProject accession PRJNA622974. The SS4–20, SS6–1 and SS15–11 raw read data are available in the NCBI Sequence Read Archives under BioProject PRJNA579424

## Supplementary Material

gkaa287_Supplemental_File

## Notes

Present address: Olga Novikova, Department of Biological Sciences, University at Albany, Life Sciences Building 2061, 1400 Washington Ave., Albany, NY 12222, USA.

Present address: John H. Starnes, Southcentral Kentucky Community & Technical College, 1845 Loop Drive, Bowling Green, KY 42101, USA.

Present address: Shouan Zhang, University of Florida/IFAS, Tropical Research & Education Center, 18905 SW 280 St., Homestead, FL 33031, USA.
