# Supplementary material for: Transposon-mediated telomere destabilization: a driver of genome evolution in the blast fungus
Source: Nucleic Acids Res. 2020 Jun 19;48(13):7197–217. doi: 10.1093/nar/gkaa287 (PMC7367193; doi:10.1093/nar/gkaa287)
Supplement: gkaa287_Supplemental_File [file gkaa287_supplemental_file.pdf]

**Table S1. Sequences of primers used in this study.**

| Primer name | Sequence (5' to 3') | Application |
|---|---|---|
| TL-F | TTAGGGTTAGGGTTAGGG | Telomere probe generation |
| TL-R | CCCTAACCCTAACCCTAA | |
| SMoter46F | TCCCGGCCAATAGACGAA | MoTeR1 RT probe generation |
| SMoter46R | CGTTAAAAGACCAGCACGAC | |
| SMoter17F | TTGGTTAACCCTGAGGATAGAA | MoTeR2 probe generation |
| SMoter17R | AATATTAACGCCGCCGTC | |
| MAGGY_Aflll-F1 | GAGGACAGGCGCCGACCG | MAGGY_AflII probe generation |
| MAGGY_Aflll-R1 | CTGTCCGTCAGTCTCGGGG | |
| MAGGY_Sacl-F1 | GGGATGCCGCTTTCGCCG | MAGGY_Sacl probe generation |
| MAGGY_Sacl-R1 | CCGCGGTATGTCGGGTTGGG | |
| MoTERnR | GTACGACAAAACCCTTAGC | MoTeR 3' junction sequencing |
| MoTER17endF | ATAGATTGGCAACAAAAAGCTACGC | MoTER1&2 (3’ amplification) |
| MoTER17endR | AAACCCGAAGGGTTCCCAAG | |
| JJEM46F2 | AAATGCCCACTAATAGAAACGG | MoTER1 (5' amplification) |
| JJEM46R | GGTAACGTCCCTATTTTTGTCTTTG | |
| JJEM17R | CCAACCCAATATATATTCCGATAGG | MoTER2 (5' amplification) |

| | | |
|---|---|---|
| JJEM17F | CAACCCCCCTGTCCAACAG | MoTER1&2 (3' amplification) |
| LpKy46t6p5-F1 | TCTTGGAGAGTTTGCCGTTC | TLP4 probe generation |
| LpKy46t6p5-R1 | GCCTTGCTTTGCAGACTCG | Figs. 6 & S15. |
| LpKy46t7p1-F1 | GCCTTGCGGAGGAGCTGC | TLP7 probe generation |
| LpKy46t7p1-R1 | CCCAATCCCGCAGAGCAGC | Fig. S4 |
| ss46t7p17-F2 | CAGGTATTGTGGCTGCTGGAGCT | TLP10 probe generation |
| ss46t7p17-A1 | TTGCCTGCTCGACTGTTCCGG | Fig. S14 |
| 31B002F | TGGTAAATATACTGGGCGAC | TLP14 probe generation |
| 31B002R | TGCCTTGTATTTCTACCGAC | Fig. 5 |
| ss7_t5_F1 | GTGACATCTTTTCGGCGGTGG | TLP-B/D probe generation |
| ss7_t5_R1 | CACCTGTTGGCATATTGCGG | Fig. S7 |
| ss108_F1 | CACCAAAATGCTTAGCCACCCC | TLP-C probe generation |
| ss108_R1 | CACAGACTCGCACCCAGGC | Fig. S8 |

**Table S2. rDNA truncations identified in this study**

| Culture ID | Telomere position[A] | TRF length[B] | Method | Telomere seed[C] |
|---|---|---|---|---|
| LpKY97 (parental) | 7581 | 2.0[D] | shotgun cloning | TAGGCT**TAGGGTTAG** |
| LpKY97 | 8325 | 2.7 | Illumina reads | CTCGTAGG**GTTAGGG** |
| LpKY97 | 3080 | 6.0 | Illumina reads | AGACTT**AGGGTTAGG** |
| 1G3SS6 | 766 | 3.6 | shotgun cloning | GGCTC**AGGGTTAGGG** |
| 2G3SS2 | 3767 | 7.6 | targeted cloning | CAGAACT**TAGGGTTA** |
| 2G3SS4 | 2670 | 5.7 | targeted cloning | GGAATA**GGGTTAGGG** |
| 2G3SS5 | 3331 | 6.2 | targeted cloning | AAAACC**TTAGGGTTA** |
| 3G1SS56 | 7067 | 1.6 | targeted cloning | TTTTTT**AGGGTTAGG** |
| 2G4SS4-20 (parental)[E] | 2276 | 5.1[D] | MinION reads[F] | CTCTTA**GGGTTAGGG** |
| 2G4SS4-20 | 924 | 3.8 | MinION reads | TCTCTTA**GGGTTAGG** |
| 2G4SS4-20 | 1188 | 4.0 | MinION reads | CATTAG**GGTTAGGGT** |
| 2G4SS4-20 | 1294 | 4.1 | MinION reads | TTGTCAG**GGTTAGGG** |
| 2G4SS4-20 | 1698 | 4.5 | MinION reads | TTGTCT**TAGGGTTAG** |

| | | | | |
|---|---|---|---|---|
| 2G4SS4-20 | 1902 | 4.7 | MinION reads | GCCCGG**TTAGGGTTA** |
| 2G4SS4-20 | 2829 | 5.7 | MinION reads | CCGAGT**TAGGGTTAG** |
| 2G4SS4-20 | 3197 | 6.0 | MinION reads | TTATAG**GGTTAGGGT** |
| 2G4SS4-20 | 4365 | 7.2 | MinION reads | GGGTTC**TTAGGGTTA** |
| 2G4SS4-20 | 5082 | 7.9 | MinION reads | GAGCTT**AGGGTTAGG** |
| 2G4SS4-20 | 5241 | 8.1 | MinION reads | CTGTTA**GGGTTAGGG** |
| 2G4SS4-20 | 5350[G] | 8.2 | MinION reads | ?[H] |
| 2G4SS4-20 | 6060[G] | 0.5 | MinION reads | ?[H] |
| 2G4SS4-20 | 6725 | 1.2 | MinION reads | GGTC**TTAGGGTTAGG** |
| 2G4SS4-20 | 7764 | 2.2 | MinION reads | TCCTCGT**TAGGGTTA** |
| 2G4SS4-20 | 8119 | 2.5 | MinION reads | TGGCGG**TTAGGGTTA** |
| 2G4SS6-1 (parental)[E] | 3331 | 6.2[D] | MinION reads | AAAACC**TTAGGGTTA** |
| 2G4SS15-11 (parental)[E] | 7581 | 2.0[D] | MinION reads | TAGGCT**TAGGGTTAG** |
| 2G4SS15-11 | 2414 | 5.3 | MinION reads | GAATCT**AGGGTTAG** |

[A] Relative to start of rDNA reference sequence (NCBI # AB026819)

[B] Approximated due to variable telomere lengths

[C] Sequence of de novo telomere junction. Putative seed nucleotides for telomere addition are underlined. First nucleotides of newly added telomere sequence are shown in bold.

[D] TRF is visible in Fig. S1.

[E] 2G4SS4-20 had 79 rDNA-telomere reads; SS6-1 had 10 and SS15-11 had 17.

[F] Novel rDNA-telomere junctions were only "called" if: i) an extended telomere repeat was present at the start (CCCTAA strand) or the end (TTAGGG strand) of the read; and ii) the rDNA blast alignment extended to within 10 bp of the telomere junction.

[G] Telomere position approximated due to unclear telomere boundary in MinION read(s).

[H] Sequence of telomere seed undetermined due to poor sequence quality at boundary.

Table S3. MAGGY insertion positions

| Single Spore ## | Insertion position (Relative to Telomere) | MAGGY orientation[A] | MoTeR target[B] |
|---|---|---|---|
| 0[C] | 3505 | + | M1 |
| 50 | 2799 | + | M1[D,E] |
| 56/145 | 854 | + | M1/M2[D,E] |
| 72 | 4205 | - | M1 [E] |
| 61 | 2265 | - | ? |
| 80 | 2860 | + | M2[F] |
| 108 | 4565 | - | M2[F] |
| 109 | 4565 | - | M2[F] |
| 115(1) | 4160 | + | M2[F] |
| 115(2) | 5866 | + | M2[F] |
| 120 | 5665 | - | M2[F] |
| 2G4SS15-11 | 1333 | - | M1[G] |
| 2G4SS15-11 | ?[H] | + | M1[G] |

[A] orientation is defined relative to MoTeR orientation

[B] M1; MoTeR1 and M2; MoTeR2

[C] Insertion present in starting culture

[D] insertion occurred in 2nd element in the tandem array with M2 in distal position (see Fig. 4A)

[E] determined by cloning

[F] determined by Southern hybridization

[G] determined from MinION sequence reads

[H] unknown because read did not extend to telomere

Table S4. *De novo* telomere additions to 5’-truncated MoTeR insertions

| Telomere | Element | Truncation position | Figure | Telomere seed[A] |
|---|---|---|---|---|
| unknown | tMoTeR1 | 784 | S3A.i | GATG**TTTGGGT** |
| TEL1 | tMoTeR1 | 2252 | S3B.i | AATAT**TAGGGT** |
| TEL5 | tMoTeR2 | 961 | S3C.i | GGGGG**TTAGGG** |
| TEL5 | tMoTeR2 | 1581 | S3C.i | TGTTTAGGG**TTA** |
| TEL7 | tMoTeR1 | 362 | S4B.iii | CGCTTTT**AGGGT** |
| TEL7 | tMoTeR1 | 782 | S4B.iii | CGCGCGATAGGG |
| TEL7 | tMoTeR1 | 4972 | S4B.iii | TAAAA**GGGTTA** |
| TEL-C | tMoTeR1 | 4622 | S8B.i | GGACCTTA**GGG** |
| TEL-C | tMoTeR1 | 4897 | S8B.i | TGGGGTTA**GGG** |
| TEL-C | tMoTeR2 | 1012 | S8B.i | TCGGG**TTAGGG** |
| TEL-A | tMoTeR1 | 1811 | S10A.i | GGCCC**TTAGGG** |
| TEL14 | tMoTeR1 | 4773 | S11.i | AAAAC**TTAGGG** |
| TEL6 | tMoTeR1 | 2940 | S12A.i | **TA**TTAGGG**TTAG** |
| TEL2 | tMoTeR2 | 1051 | S13A.i | AAATC**TTAGGG** |
| TEL2 | tMoTeR1 | 1452 | S13A.i | TCAAC**TTAGGG** |
| TEL2 | tMoTeR1 | 4728 | S13A.i | AAA**GGGTTAGG** |
| TEL12 | tMoTeR1 | 4894 | S13B.i | TATAA**GGGTTAG** |
| TEL12 | tMoTeR1 | 3910 | S13B.i | CTAGGGTT**AGGG** |
| TEL12 | tMoTeR1 | 4622 | S13B.i | GGACC**TTAGGG** |
| TEL10 | tMoTeR1 | 4731 | S14B.1 | AAACA**TTAGGG** |
| TEL10 | tMoTeR1 | 279 | S14B.i | CTAGG**GTTAGGG** |
| TEL10 | tMoTeR1 | 265 | S14B.i | CAAGGG**TTAGGG** |

| | | | | |
|---|---|---|---|---|
| TEL10 | tMoTeR1 | 4610 | S14B.i | AAA<u>TTA</u>**GGGTTA** |

[A] Putative telomere seed sequences at MoTeR 5' ends are underlined; added telomere sequence is in bold.

Table S5. MoTeR relics in the LpKY97 genome.

| Chromosome position | Sequence of MoTeR 3' end plus flank[A] |
| --- | --- |
| **Chr1**, 6,426,343 bp, no relics | |
| **Chr2**, 7,917,642 bp, no relics | |
| 25940 | **ACGCGAATTAAAA**CCCTAAGACCTATTATATCGGTCGAAAAAATCGCGGG |
| 42376 | **GCGCGAATTAAAA**CCCTAACCCTAACTAAATTTAATTCGCCAAATAAACT |
| **Chr3**, 7,435,242 bp | |
| 152722 | TAATCACGTTGGTTAACGTGACCTTTGTGGGTTAGGG**TTTTAATTCGCGT** |
| 2548296 | ACGCCGCAACGGCCATCAAATTAAGTCAGGGTTAGGG**TTTTAATTCGCGT** |
| 2742283 | **ACGCGAATTAAAA**CCCTAACCCTACAAGTTAGGAATGCGGCTGTTTATAG |
| 7355697 | CGTGAAAGCCATGGCTTGCTTTCAACAGGTCTTAGGG**TTTTAATTCGCGC** |
| 7414869 | ACCAAAGCCGTTTAAAATCCAATGGCGCCGCTTAGGG**TTTTAATTCGCGC** |
| **Chr4**, 5,421,781 bp, no relics | |
| **Chr5**, 4,407,506 bp | |
| 5248 | **GCGCGAATTAAAA**CCCTAACCCTAAGACTTGAATTCCTGCTCGTATTTTC |
| 4395439 | **GCGCGAATTAAAA**CCCTAACCCGTGCCGGCTCGGCATAACTTTCTTCCGG |
| 4397484 | TTTTTATTTTATTTTTATTTTTATTTTAGGGTTAGGG**TTTTAATTCGCGC** |
| 4397781 | TAAAAACAAAATAAAAACAAAAACATTAGGGTTAGGG**TTTTAATTCGCGC** |
| **Chr6**, 6,132,319 bp | |
| 83149 | **ACGCGAATTAAAA**CCCTAAGGGAATTAATAAAGGGCCGGCGCGCCGCGCC |
| 6123856 | GTAGGTTGGGGTTATAAGGTTAGGGTTAGGGTTAGGG**TTTTAATTCGCGC** |
| 6124982 | GTTAAGCCATTTTCGACCTAAATAACTAGGGTTAGGG**TTTTAATTCGCGC** |
| 6125449 | GCAATGGAAATTGCTGCCGTTGTTCTGGACCTTAGGG**TTTTAATTCGCGC** |

| | |
|---|---|
| **Chr7**, 3,850,492 bp | |
| 3843724 | CAAAAACAAAACCAAAAACAAAAACGAAAACTTAGGG**TTTTAATTCGCGC** |
| **MiniChr1**, 299,4574 bp | |
| 138552 | **ACGCGAATTAAAA**CCCTAAGTAAGCTTTCTCCTCGGCAAAGGCATTTGTC |
| 398360 | GACAAATGCCTTTGCCGAGGAGAAAGCTTACTTAGGG**TTTTAATTCGCGT** |
| 1359742 | TGTTTAAACGCCTGTACGCTTGGGATTAGGGTTAGGG**TTTTAATTCGCGT** |
| 1362129 | TGTTTAAACGCCTGTACGCTTGGGATTAGGGTTAGGG**TTTTAATTCGCGT** |
| 1364517 | TGTTTAAACGCCTGTACGCTTGGGATTAGGGTTAGGG**TTTTAATTCGCGT** |
| 1366906 | TGTTTAAACGCCTGTACGCTTGGGATTAGGGTTAGGG**TTTTAATTCGCGT** |
| 2383001 | **CCGCGAATTAAAA**CCCTAACGCAGCAGTCAGTGGGCGTCTTACAGAGCAG |
| 2783081 | GGACCGATAAACGAACCTAAAACCTTAAACCTTAGGG**TTTTAATTCGCGT** |
| 2806591 | TTAGGCTCCGTTAGCGGCCATTTATTTAGGGTTAGGG**TTTTAATTCGCGT** |
| **MinChr2**, 912,782 bp, no relics | |

[A] MoTeR 3' end is in bold; telomere repeat is underlined. Relic sequences on left are in + orientation; those on right are reverse-complemented.

Table S6. Rearrangements identified in this study

| Rearrangement type | Telomere(s) affected | # of events identified[A] |
|---|---|---|
| rDNA truncations | 1 | 24 |
| Replication slippage in TTTGGG/TTAGGG repeats | 3, 6, 7, 11, 12, 14 | 21 |
| Intra-/unequal sister chromatid exchange; gene conversion | 4, 6, 8, 10, A | 14 |
| IT break + heal | 6, 12, 14, A, B, D | 13 |
| MAGGY transposition | 11, A & unknown[B] | 11 |
| BIR via D-loop formation | 3, 4, 7, 12, C | 7 |
| IT break + ectopic invasion | 2, 7, 14, B | 7 |
| MoTeR acquisition | 1, 4[C], 5, 7, 8[C], B | 7 |
| Terminal truncation | 7, 10 (strain 2539) | 3 |
| IT break, MoTeR truncation + *de novo* telomere form. | 2, 10 | 1 |
| Dupl. of internal sequence | unknown | 1 |
| **Total** | | **109** |

[A] Independent rearrangements

[B] Identified using Southern blot data (Fig. 4)

[C] Read lengths were too short to allow resolution of TEL4 from TEL8

SUPPLEMENTAL FIGURE LEGENDS

**Fig. S1. Single-spore generations used in this study.** Note: populations used in this study are shown in gray boxes. *Experiment 1*. First generation spores of LpKY97 were harvested from oatmeal agar and used to inoculate plants of annual ryegrass cultivar Gulf. Second generation spores were harvested from lesions and used to inoculate a second batch of plants and a set of 10 single spore (SS) cultures was established. Third generation spores were collected from lesions and 10 third generation SS cultures were obtained. *Experiment 2*. A single spore of LpKY97 was used to inoculate an oatmeal agar plate and the resulting first spore generation was used to initiate two rounds of infection on perennial ryegrass cultivar Linn. Twenty SS isolates were established at each generation. Permanent stocks of several third generation, SS cultures were reactivated on oatmeal agar and up to 20 fourth generation SS cultures were generated. *Experiment 3*. The original LpKY97 SS culture generated in experiment 2 was serially cultured on complete medium agar by allowing it to grow across the full diameter of two 85 mm complete agar plates and 250 SS cultures were established.

**Fig. S2. Telomere-rearrangements arising after successive generations of *in planta* growth, starting with a culture that originated from a single, genetically-purified spore.** Telomeric restriction profiles of SS isolates from successive rounds of plant infection (Fig. S1A). DNA samples were digested with *Pst*I, fractionated by gel electrophoresis, blotted to a membrane and hybridized with a telomere probe. The open arrowhead marks the highly unstable “rDNA” telomere (TEL1), while the filled arrowhead marks a stable telomere that rarely exhibited rearrangement (TEL5). Novel telomeric restriction fragments that were cloned characterized and described here are labeled "a", "f", and "n." Black dots highlight rearranged rDNA telomere fragments. The asterisk marks a novel TRF that is shared by culture 5, 6, 8 and 9 and which

arose through a MoTeR2 insertion in TEL-C - presumably via a transposition event (see Fig. S8 variant ii).

**Fig. S3. Target site analysis of putative MoTeR transposition events.** A) i. Putative tMoTeR1 transposition event giving rise to TRF-d; ii. Sequence alignment showing tMoTeR insertion site pre- and post-insertion. B) i. Putative tMoTeR1 transposition event giving rise to TRF-e; ii. Sequence alignment of insertion site pre -and post-MoTeR insertion. C) i. Putative tMoTeR2 transposition events giving rise to TRF-f; ii) Sequence alignment of insertion site pre -and post-MoTeR insertion. MoTeR truncation positions are listed in parentheses.

**Fig. S4. TEL7 rearrangements identified using a telomere-linked probe.** TEL7 was cloned on a 10.5 kb *Pst*I fragment. A Southern blot of *PstI*-digested SS DNAs was sequentially hybridized using the telomere and TLP7 probes. TEL7 variants are labeled i, ii, iii and iv. The asterisk is to highlight a TRF that is believed to correspond to variant iv but a definitive link has not yet been established; B) Structures of the four variants as determined from MinION reads. All structures were inferred from individual MinION reads that spanned the entire distance from the chromosome unique sequence to the telomere. In most SS cultures, the TLP7 probe hybridized to fragments with a molecular size of ~10.5 kb (bands i and ii). Isolates SSs 12, 13, 17 and 18 had signals at a position corresponding to ~ 16 kb (band iv), SS4 had one slightly larger than 16 kb (band iii), and the 2G1SS1 parent culture had a signal at ~13 kb.

The MinION reads for 2G4 SSs 6-1 and 15-11 revealed that the 10.5 kb TRF - which was also present in the original LpKY97 culture (see Fig. 1) but not 2G1SS1 (this figure, panel A) - had a single, full-length MoTeR1 insertion in the telomere with a short interstitial telomere repeat $(TTAGGG)_2$ (i & ii). In contrast, most reads from SS4-20 identified a TEL7 variant with four truncated MoTeR copies distal to the intact element (see panel B.iii). These four elements

were duplicates of truncated variants found at the end of TEL-D (Fig. S5). Considering that the parental telomere lacks obvious break-inducing features, we hypothesize that the first three elements were acquired through a multi-step process. Step 1 likely involved break-induced replication of a composite MoTeR cassette from TEL-D; in step 2, a terminal duplicate of these last three elements was then generated by D-loop formation & extension; step 3 involved terminal resection that eliminated the two distal tMoTeR copies; and, finally, step 4 involved healing of the TTAGGG seed at the tMoTeR2:tMoTeR1 junction. Under this scenario, the slightly shorter variant with just two truncated elements distal to the intact MoTeR1 could be the product of more extensive resection prior to healing (iv). The low abundance of variant (iv) in SS4-20 (and its similar size) is probably why a corresponding band is not visible in the SS4-20 lane but it likely corresponds to band "iv" seen in SS 12, 13, 17 and 18. We were unable to characterize the ~13 kb TRF in LpKY97 but given that it is slightly smaller than variant "iv," it possibly comprises an array with a telomere-healed break at the $(TTAGGG)_3$ repeat.
Summary of inferred rearrangement mechanisms: i -> iii) acquisition of a composite MoTeR cassette comprising the three distal elements in TEL-D (see Fig. S5), followed by acquisition of a truncated MoTeR2 into the newly acquired telomere; iii -> iv) as above, but with resection/attrition and telomere healing as an alternate pathway to the second MoTeR2 acquisition.

**Fig. S5. A single MoTeR array with a MAGGY insertion in the LpKY97 parental strain.** The combined MinION assembly identified a single MAGGY insertion in a complex MoTeR array in TEL-D on minichromosome 2. Note: the entire array was captured in a single MinION read thereby confirming the array's structure.

**Fig. S6. STR contractions at TEL8 and stable telomere structures identified among MinION reads.** Unless otherwise stated, telomere instability was documented by interrogating only those reads that started in the chromosome-unique region proximal to the telomere and terminated in telomere repeats (or *vice versa*). In other words, they spanned entire MoTeR/subtelomeric repeat arrays (note: the C-rich strand of the chromosome end was rarely captured via MinION sequencing). A) Alterations of the STR subtending TEL8. This telomere has the same subtelomeric tandem repeat structure as TEL4 and, not unexpectedly, it experienced the same types of alterations - namely subtelomeric array contraction/expansions, as well as MoTeR1 acquisition by a plain telomere (e.g..i & ii). Intriguingly, in both cases, the interstitial telomeres that separated the MoTeR element from the internal sequence were nine TTAGGG repeat units in length (see variant ii & Fig. 6B, variant iv). This suggests that the MoTeR sequence was exchanged between the two chromosome ends, with the most likely route being unequal, inter-chromosomal exchange, as this would also explain the reciprocal extensions and contractions. Summary of inferred rearrangement mechanisms: i) intrachromatid unequal sister chromatid exchange; i -> ii) MoTeR1 transposition. B) Structures of stable telomeres. The organization of the most terminal *Pst*I fragments was determined by subcloning and sequencing (TEL5), and/or by analysis of MinION reads (TELs 5, 9 and 13). No major structural variants were detected for TELs 9 and 13, although the numbers of variant TTTGGG repeats was different in some reads. Note the absence of long interstitial telomeres, and the presence of TTCGGG/TTTGGG repeats subtending the telomere proper.

**Fig. S7. Telomere rearrangements at minichromosome TELs B and D identified via Southern blotting and MinION sequencing..** A) Structural organization of TRF-B variants showing the location of TLP-B. Targeted cloning of TRFs from 2G3SSs 2, 4, 5, 6, and 7 resulted

in the isolation of an 8.3 kb terminal fragment that contains a single MoTeR1 insertion in TEL-B on mini-chromosome 1 (variant B.iv). B) Structural organization of TEL-D variants. The subtelomere region with sequence identity to TEL-B is highlighted using a gray-shaded background. C) Southern hybridization analysis of SS DNAs with the telomere probe and TLP-B. A blot of *PstI*-digested SS DNAs was sequentially hybridized using the telomere and TLP-B probes, as shown below the respective phosphorimages. TLP-B produced three main hybridization signals at 8.3 kb, ~11 kb and >12 kb (right-hand panel). The 11 kb band remained unaltered in all SS cultures consistent with its origination from an internal chromosome location that lacks MoTeR sequences, interstitial telomeres, or other obvious de-stabilizing features (data not shown). In contrast, both the upper and lower bands (>12 kb & 8.3 kb) showed rearrangements in certain SS isolates. The lower band exhibited varying hybridization intensities among isolates. It was very faint in SS4, and was largely replaced by a strong signal at ~16 kb, and a weaker one at ~14 kb (B.i and B.iii, respectively); and it was completely absent in 2G1SS1, which instead possessed novel bands of ~11.7 kb, 10 kb, and faint signals at ~9.5 kb, and a high molecular weight fragment (right-hand panel). The largest hybridizing fragment (D.i) contains TEL-D and was absent in SSs 5, 6, 8 and 9 which, instead, showed doubly intense bands at ~8.3 kb (D.ii*).

Inspection of the MinION assembly of SS4-20 revealed that the original 8.3 kb TRF, which contained a single MoTeR1 insertion (panel A, variant iv), was a truncated version of a longer TRF that contained a multi-MoTeR array with an extended interstitial telomere distal to the aforementioned MoTeR1 (panel A, variants i, ii and iii). Normally, the fact that the majority of SS isolates possessed variant B.iv would imply that this short form is the original parental structure. However, given the absence of obvious destabilizing features, it is more likely that the

shorter TRF-B forms were derived from longer variants via breakage and healing of the interstitial telomeres at the MoTeR junctions. The absence of variant B.iv in SSG1SS1 is consistent with this interpretation, and it appears that the most of the SS isolates might have inherited a truncated variant that arose in the original colony while it was being grown to generate spores for the first round of plant infection. The structure of variant B.v is most easily explained as a result of MoTeR2 insertion at the internal TTAGGG motif in the B.iv telomere [i.e., TTCGGG(TTTGGG)$_5$TTAGGGTTTGGG(TTAGGG)$_{28\text{-}33}$]. In this case, the additional TTAGGG repeats found in the newly-created interstitial telomere would have been contributed by the invading MoTeR. Alternatively, the interstitial telomere could have become altered through replication slippage.

The MinION assemblies revealed that TEL-D contains a complex MoTeR array spanning a total of 48 kb, and comprises 18 separate tMoTeR1 and tMoTeR2 insertions (Fig. S5). One of the tMoTeR1 copies is interrupted by a MAGGY insertion (panel B, variant D.i) which explains why the 12kb+ hybridizing band is not telomeric (panel C, left-hand blot). Inspection of MinION reads for the SS culture 6-1, which lacked the 12kb+ signal, revealed a D.ii variant that is very similar in size to the B.iv fragment. D.ii likely arose following a break at the interstitial telomere tract, (TTAGGG)$_3$, which is distal to the most proximal tMoTeR1 copy, with the break having been subsequently healed by telomere addition (panel B.ii). The original tMoTeR1 truncation boundary (position 362) was conserved, indicating that at least a portion of a TTAGGG repeat at the breakpoint was retained and served as a seed for telomere healing. Interestingly, the complete absence of reads spanning the MoTeR1/MAGGY junctions indicates that the sequences beyond the truncation point were eradicated from the SS6-1 and SS15-11 genomes. Summaries of inferred rearrangement mechanisms, **TEL-B**: i -> ii) Interstitial telomere break & healing; i or ii

-> iii) Interstitial telomere break & healing; i or ii or iii -> iv) Interstitial telomere break & healing; iv -> v) MoTeR2 transposition; **Tel-D**: i -> ii) Interstitial telomere break & healing. All structures were inferred from individual MinION reads that spanned the entire distance from the chromosome unique sequence to the telomere.

**Fig. S8. TEL-C rearrangements identified via Southern blotting and MinION sequencing.** A) Southern hybridization analysis of SS DNAs using the telomere probe and TLP-C. A blot of *PstI*-digested SS DNAs was sequentially hybridized using the telomere and TLP-C probes, as shown below the respective phosphorimages. Targeted telomere cloning with DNA from 2G3SSs 4 and 6 resulted in the recovery of plasmids with inserts of ~ 7.6 and 9.3 kb, respectively. These clones represented two variants of TEL-C on mini-chromosome 2. As expected, the TLP-C probe bound primarily to two loci in genomic DNA from the SS isolates. One, represented by a ~7.0 kb fragment, corresponded to the internal locus and showed no rearrangement, while the other exhibited alternate forms with molecular sizes approximating 7.6 and 9.3 kb. These latter fragments corresponded to the two cloned variants, with the shorter TRF (i) being the predominant form. Cultures 5, 6, 8 and 9 all exhibited the larger TRF (ii) (panel A, right-hand panel), consistent with the clonal relationship evidenced by their telomere profiles (left-hand panel). Initially, we suspected that the 9.3 kb TRF arose via transposition. However, this is hard to reconcile with the organization of the telomere-like tracts flanking the newly-acquired MoTeR2. Instead, it appears that the element was acquired via a BIR event that was initiated as a result of telomere de-protection. Based, on the MoTeR2 flanks, it appears that the element was acquired from the most distal position in the MoTeR array in TEL-6 (Fig. S12A, variant ii or v). B) Structures of TEL-C variants recovered by targeted cloning of TRFs from SS cultures 2G3SS4 and 6 (see right-hand panel, bands i & ii). All of the MinION reads from SS

isolates 4-20 and 15-11 were consistent with the structure of the 7.6 kb TRF and there was no evidence for the emergence of other variants within the SS cultures that possessed this form (panel B.i). Likewise, the TEL-6 fragment was visible in ~150 additional SS cultures. Consequently, aside from the one alteration recovered in SS6 (and its sister cultures), this TRF appears to be generally quite stable. No further rearrangements were detected among the small number of TEL-C reads from SS6-1. However, based on the presence of an extended interstitial telomere between the two intact MoTeR copies, we predict that new breaks should promote further alterations at an appreciable frequency. Summary of inferred rearrangement mechanisms: i -> ii) Telomere deprotection followed by BIR using the most distal MoTeR2 in TEL6 as a template. Structures were inferred by characterizing cloned TRFs and by inspecting individual MinION reads that spanned the entire distance from the chromosome unique sequence to the telomere.

**Fig. S9. Telomeric restriction fragments representing *de novo* MAGGY insertions into MoTeR sequences.** MoTeR segments are shaded as follows: white = present in MoTeR1; light gray = unique to MoTeR2; black = 3' sequence shared by both elements. MAGGY LTR sequence is shown as a box with an arrow underneath indicating orientation, and the insertion positions are shown in parentheses.

**Fig. S10. *De novo* MAGGY insertions in TEL-A and TEL11 identified in MinION reads.** Unless otherwise stated, all structures were inferred from individual MinION reads that spanned the entire distance from the chromosome unique sequence to the telomere. A) Structural variants of TEL-A found among the MinION read data for the three SS isolates. The TRF terminating in TEL-A had a highly variable structure with six different variants being identified. The majority of reads had a truncated MoTeR1 in the most proximal array position with an intact MoTeR1

adjacent to it, and variation being limited to distal composition. TEL-A.i had a MoTeR2 in the distal position, A.v had a second intact MoTeR1, while A.ii was simply capped with a telomere. The A.ii form was likely derived from A.i simply by breakage and healing. On the other hand, the A.v variant appears to have originated through recombination with another telomeric array, causing acquisition of the characteristic $(TTTGGG)_{10}TTAGGGTTTGGG(TTAGGG)_2$ interstitial telomere tract that is found in other telomeres (TEL6, TEL14, TEL-C). All of the TEL-A reads from SS6-1 had a MAGGY inserted in the proximal tMoTeR1 (vi). However, none of the reads extended to the chromosome thereby precluding identification of other possible alterations. TEL-A.iii had its probable origin in an intrachromosomal, or unequal sister chromatid exchange that paired the truncated MoTeR1 with the intact copy, while A.iv has a structure consistent with an interstitial telomere break and healing of A.iii. The original parental form had a truncated MoTeR1 in the proximal position. In SS6-1, this tMoTeR1 contained a MAGGY insertion (variant A.vi). B) The parental form of TEL11 contained a single, full-length MoTeR1 insertion, which itself contained a super-insertion of a previously undiscovered retrotransposon, "RETRO8," at nucleotide position 4205. A single MinION read from SS15-11 possessed a second super-insertion, consisting of a MAGGY integrated at nucleotide position 1033. LTRs are shown as block arrows at each end of RETRO8 and MAGGY, and the elements' labels also reflect their orientations. Note the differences in variant telomere tracts among the different MinION reads. Summaries of inferred rearrangement mechanisms - **TEL-A:** i -> ii) Interstitial telomere break & healing; i -> iii) intrachromatid recombination between MoTeR1 and tMoTeR1 copy; iii -> iv) Interstitial telomere break & healing; ii -> v) ectopic recombination; i or ii or v -> vi) MAGGY insertion; **TEL11**: length differences in variant telomere tracts in i, ii, iii, & iv) replication slippage; ii -> v) MAGGY insertion.

**Fig. S11. TEL14 variants identified among MinION reads.** All structures were inferred from individual MinION reads that spanned the entire distance from the chromosome unique sequence to the telomere. Note the differences in variant telomere tracts at MoTeR junctions and subtending the telomeres proper. Inferred rearrangement mechanisms: i -> ii interstitial telomere break, repair via unequal sister chromatid exchange, replication slippage; i or ii -> iii) interstitial telomere break, repair via unequal sister chromatid exchange; i or ii to iv) interstitial telomere **break plus slippage; i or ii or iii or iv -> v) interstitial telomere break & healing; i or ii to vi)** interstitial telomere break, repair via unequal sister chromatid exchange.

**Fig. S12. Structural variants of TEL6 identified using MinION reads.** All structures were inferred from individual MinIO**N reads that spanned th**e entire distance from the chromosome unique sequence to the telomere. Note the highly unusual variant telomere tracts separating the MoTeR copies and subtending the telomere proper. Unless otherwise stated, telomere instability was documented by interrogating only those reads that started in the chromosome-unique region proximal to the telomere and terminated in telomere repeats (or *vice versa*). A) The MinION reads containing TEL6 revealed the presence of multiple MoTeR1 and MoTeR2 insertions, with most molecules harboring between five and 10 element copies. Several of these MoTeR arrays were unusual in having abnormally long and complex interstitial telomeres comprising alternating runs of the various TTAGGG-like motifs (e.g., variants iii - vi); and in two cases, the telomere proper was subtended by variant repeats with even more complex organizations (6.iv & 6.vi). Variants i and ii had a truncated tMoTeR1 as the most proximal array element, which was replaced by an intact MoTeR1 in all other forms. B) Possible mechanisms giving rise to TEL6 variant ii. Variant i could have given rise to variant ii through intrachromatid recombination, or an unequal sister chromatid exchange, involving MoTeR1 copies 2 and 4. C) B) Possible

mechanisms giving rise to TEL6 variant iii. This variant is the likely product of intrachromatid recombination, or an unequal sister chromatid exchange, between the proximal tMoTeR1 and MoTeR1 copy 2 Fig. S15C). Variant 6.iv almost certainly arose from 6.iii via interstitial telomere breakage and healing; and it seems likely that 6.vi was generated from 6.iii, 6.iv or 6.v by the same mechanism. On the other hand, 6.v was probably derived from 6.iii through an intrachromatid or unequal sister chromatid exchange between MoTeR1 copies 3 and 4. Summaries of inferred rearrangement mechanisms: i -> ii & i -> iii) intrachromatid/unequal sister chromatid exchange & slippage (see panels B and C for examples); iii - iv) interstitial telomere break and healing & slippage; iii or iv or v -> vi) interstitial telomere break and healing & slippage; i or ii or iii -> v) intrachromatid/unequal sister chromatid exchange & slippage.

**Figure S13. Structural variants of TEL2 and TEL12 identified using MinION reads**. Unless otherwise stated, telomere instability was documented by interrogating only those reads that started in the chromosome-unique region proximal to the telomere and terminated in telomere repeats (or *vice versa*). A) Inspection of MinION reads showed that TEL2 was contained on a *Pst*I fragment of between ~46 kb and 48 kb and the subterminal sequences comprise another tandem repeat array. Note that this array is disqualified from being classified as a "subtelomeric" tandem repeat due to its presence at a single chromosome end (see "Terminology"). In SS4-20 and SS6-1, the telomere contained a truncated MoTeR2 and an intact MoTeR1, with short interstitial repeats (≤3 TTAGGGs) (i & ii). Differences in the sequence of variant repeats subtending the telomere suggests that the TEL2.ii form was derived from TEL2.i via replication slippage. In SS15-11, a truncated MoTeR1 was found inserted between the tMoTeR2 and MoTeR1 copies and two different terminal organizations were identified (iii & iv). The newly acquired tMoTeR1 was truncated at the same position as two copies found in TEL4 (Fig. 6),

suggesting that it was acquired via gene conversion, or that TEL2 and TEL4 underwent an exchange of sequence. These scenarios also account for the acquisition of the drastically truncated MoTeR1 that is found in a terminal position in TEL4. Variant TEL2.iv presumably arose from TEL2.iii through an interstitial telomere break, followed by resection and, eventually, *de novo* telomere formation. B) Most reads containing TEL12 supported a telomere structure comprising insertions of three truncated MoTeR1s in a proximal position and intact, distal copies of MoTeR1 and MoTeR2, with long interstitial telomere/variant repeats between them (i & ii). Some reads revealed a shorter TTAGGG tract subtending the telomere (7 TTTGGG repeats vs. 10), consistent with replication slippage. Variant 12.iii likely arose from 12.ii via breakage and healing, while variant 12.iv appeared to have originated via D-loop formation and extension - which best explains the identical structure of the two distal interstitial telomeres. Inferred rearrangement mechanisms - **TEL2**: ii -> i) exchange/gene conversion with TEL-B (see Fig. S7A, variant SS4-20.iv); ii) -> iii) ectopic recombination with TEL4 (see Fig. 6B, variant SS6-1.v); i -> iv) interstitial telomere break, resection, *de novo* telomere formation; **TEL12**: i or ii -> iii) interstitial telomere break & healing; i -> iv) D-loop formation and extension. All structures were inferred from individual MinION reads that spanned the entire distance from the chromosome unique sequence to the telomere.

**Fig. S14. Unequal sister chromatid exchange at TEL10.** Targeted cloning resulted in the capture of both parental and rearranged versions of TEL10 on *Pst*I fragments of 15 kb and 11.5 kb, respectively. A) A Southern blot of *PstI*-digested SS DNAs was sequentially hybridized using the telomere and TLP10 probes. As expected, strain 2G3SS4 which yielded the clones with the 11.5 kb inserts exhibited a hybridization signal at a position corresponding to 11.5 kb, while the remaining cultures - including 2G3SS7, the strain that produced the larger insert - all had

signals at a position corresponding to ~16 kb. B) TRF10 structures identified among individual MinION reads that spanned the entire distance from the chromosome unique sequence to the telomere. The TRFs that correspond to the three variants identified among MinION reads are labeled i, ii and iii. The MinION reads revealed that the larger, parental TRF (variant 10.i) had four truncated MoTeR1s inserted tandemly in the telomere, two of which were missing the same amount of 5' sequence (264 nt). The TRF was unusual in that it also contained a second, drastically truncated MoTeR1 (MoTeR1 relic) at an "internal" location approximately 2 kb away from the telomere but whose orientation was inverted with respect to the telomeric copies. Also present was an inverted, interstitial telomere just 300 bp away from the start of the TTAGGG repeats at the MoTeR array border. It is clear that the internal relic was once telomeric because it had 1.5 telomere repeat units at its 3' end. In SS SS4-20, TRF10-1 had an identical organization, except that it possessed just one copy of the larger truncated MoTeR1 (variant ii). As was seen with TEL14 (Fig. 5), SS cultures possessing TRF10.i often exhibited signals at the same position as TRF10.ii (panel A), which suggests that 10.i also undergoes spontaneous truncations at a high frequency, that gives rise to structure 10.ii in some nuclei. However, this conclusion was not supported by the sequence data because, while two variants were identified among reads for SS15-11, their sizes were ~15 kb and > 20 kb. Furthermore, because the MoTeR array contained no long interstitial telomeres (panel B), there was no reason to suspect it would undergo recurrent breakage. Instead, the identification (and structure) of the longer (20 kb+) variant, and its presence - albeit as another faint band - in several SS cultures that possessed the short form, presented an alternative mechanism that fully explained the data. Specifically, the large and short variants are the expected products of an unequal sister chromatid exchange between the two

larger tMoTeR1 insertions. It is not clear what would promote such a high level of recombination in this array.

Summary of inferred rearrangement mechanism: i -> ii & iii) Unequal sister chromatid exchange.

**Fig. S15. Rearrangements at TEL4 identified by CHEF gel analysis**. A) Analysis of TEL4 RFs in representative SS isolates. Agarose microbead-embedded genomic DNA was digested with *Pst*I, fractionated using CHEF electrophoresis, electroblotted to a nylon membrane and probed sequentially with telomere, TLP4, MoTeR1 and MoTeR2 probes. The resulting phosphorimages are shown. B). Analysis of TEL4 RFs in the DNA samples used for MinION sequencing. High molecular weight genomic DNA was digested with *Pst*I, fractionated on a 0.4% agarose gel, electroblotted to a nylon membrane and probed with the TLP4 probe. Note: equal amounts of DNA were loaded in each lane. Molecular sizes are in kilobases.

**Figure S16. D-loop mediated MoTeR array expansions in TEL3**. A) Structures of TEL3 variants identified among MinION reads. All structures were inferred from individual MinION reads that spanned the entire distance from the chromosome unique sequence to the telomere. Numbers of reads supporting each structure are indicated in parentheses. B) Schematic showing MoTeR array extension via break-induced replication with D-loop formation. i) MoTeR2 transposition (or an interstitial telomere break at a MoTeR2 proximal junction) generates a free, variant telomere-terminated DNA end (ii); iii) free end invades interstitial telomere; DNA synthesis replicates MoTeR2 sequence (iv); end of newly synthesized strand is either healed with a telomere (v), or initiates a second D-loop (vi); etc. C) Schematic showing array extension via invasion of extra-chromosomal MoTeR circles. i) D-loop formation produces a replication intermediate that is resolved form an extrachromosomal circle. ii) a free end invades the circular molecule and is replicated through iterative cycles around the circular strand.

The inferred rearrangement mechanisms giving rise to the various structures in A are as follows: i -> ii) MoTeR2 transposition; ii -> iii -> iv -> v) BIR involving D-loop formation by free end; followed by recurrent cycles of strand invasion and extension (see B). Alternatively, resolution of the initial replication intermediate creates an extrachromosomal circle that provides a template for repeated rounds of array extension for the free DNA end (see C).

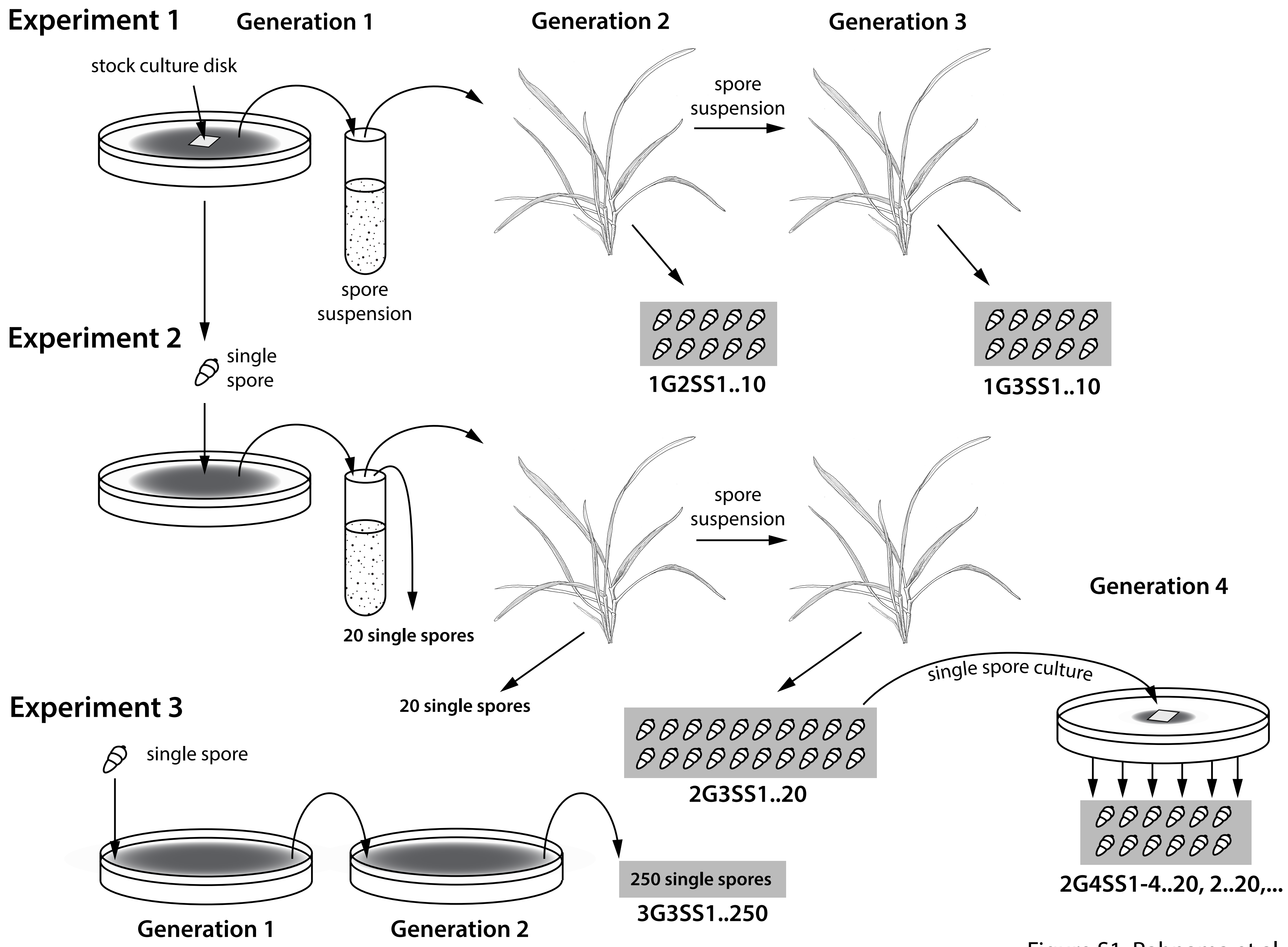


Figure S1. Rahnama et al.

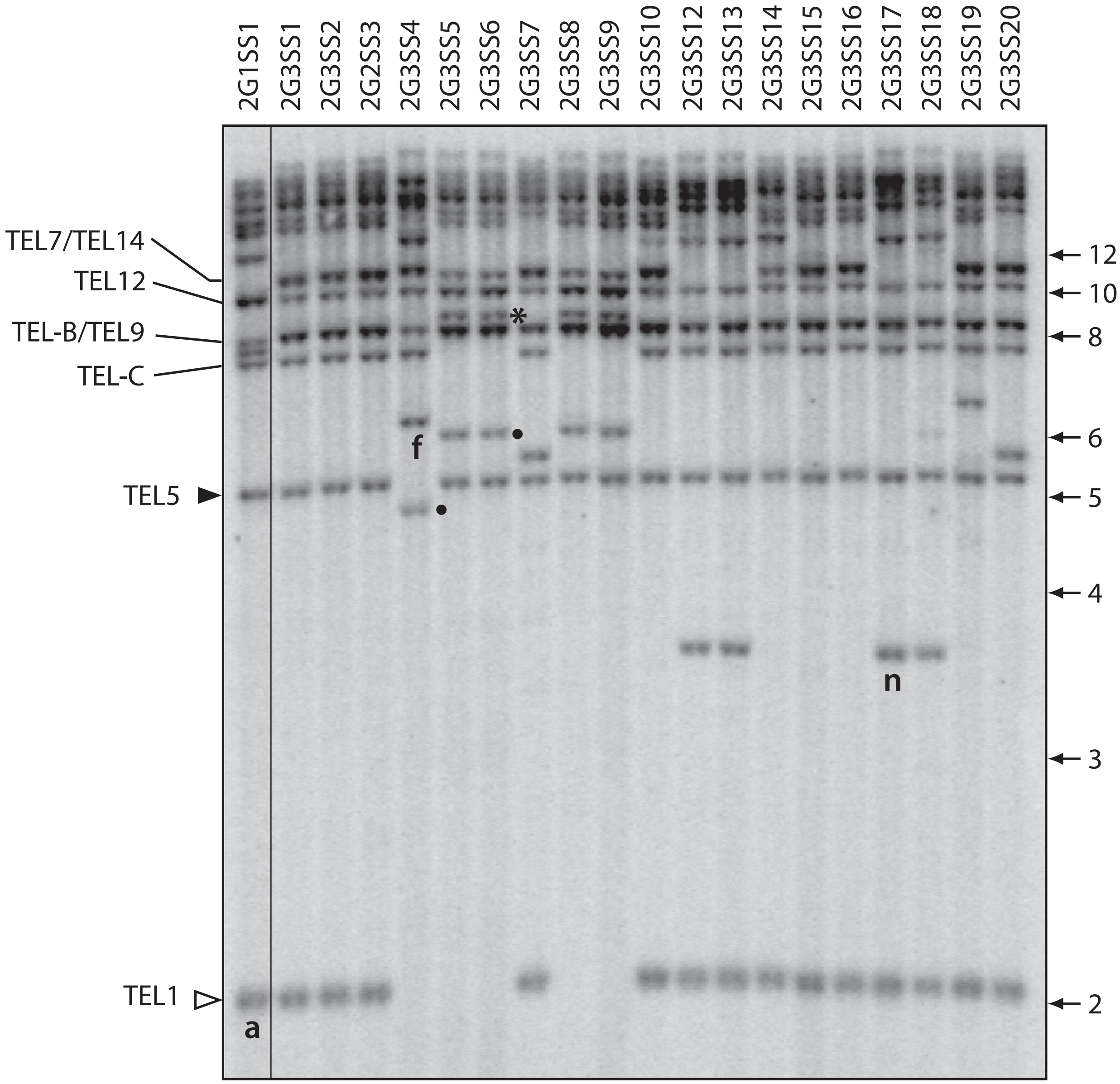


Figure S2. Rahnama et al.

**A) tMoTeR1 transposition:**

i) TRF-d

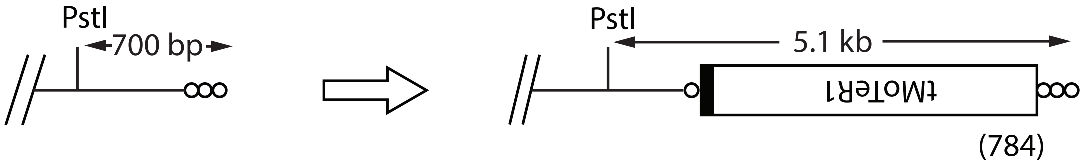


ii) AACCCCACTCCGTTAGGGTTAGGTTAGGGTTAGGGTTAGGGTTAGGGTTAGGGTTAGGGTTAGGG...

AACCCCACTCCGTTAGGGTTAGGTTAGGGTTAGGGTTAGGG**TTTTAATTCGC...GCGCGATG**TTTGGGTTAGGGTTAGGG...

3'<--------------------5' (784)

**B) tMoTeR1 transposition:**

i) rDNA telomere (TRF-e)

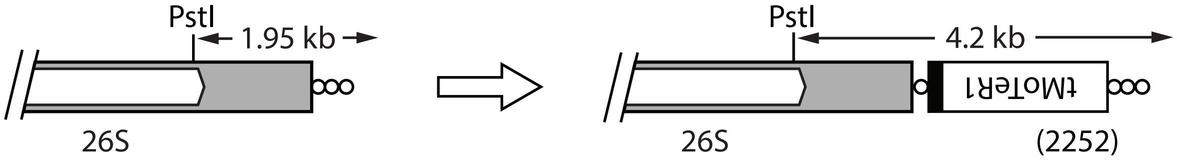


ii) CAGGGTAGGTGCAGGGTAGGCTTAGGGTTAGGGTTAGGGTTAGGGTTAGGG...

CAGGGTAGGTGCAGGGTAGGCTTAGGG**TTTTAATTCGC...GATACCGAATAT**TAGGGTTAGGGTTAGGGTTAGGG...

3'<------------------------5' (2252)

**C) tMoTeR2 transposition:**

i) TEL5 (TRF-f)

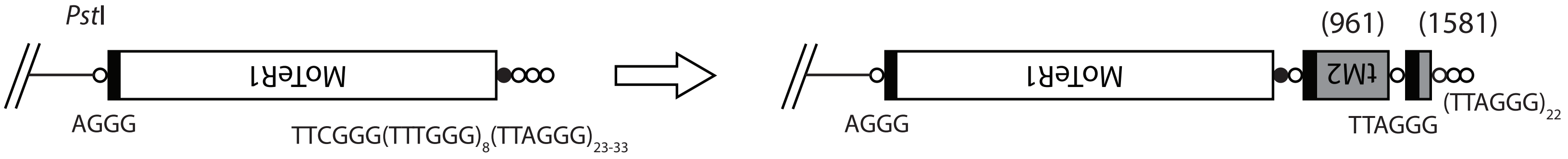


ii) TTCGGGTTTGGGTTTGGGTTTGGGTTTGGGTTTGGGTTTGGGTTTGGGTTTGGGTTAGGGTTAGGGTTAGGGTTAGGGTTAGGG...

TTCGGGTTTGGGTTTGGGTTTGGGTTTGGGTTTGGGTTTGGGTTTGGGTTAGGG**TTTTAATTCGC...GGTTTGGGGGGTT**AGGG**TTTTAATTCGC...TGGTCCTCTTT**AGGG...

3'<------------------------5' (961) (1581)

Figure S3. Rahnama et al.

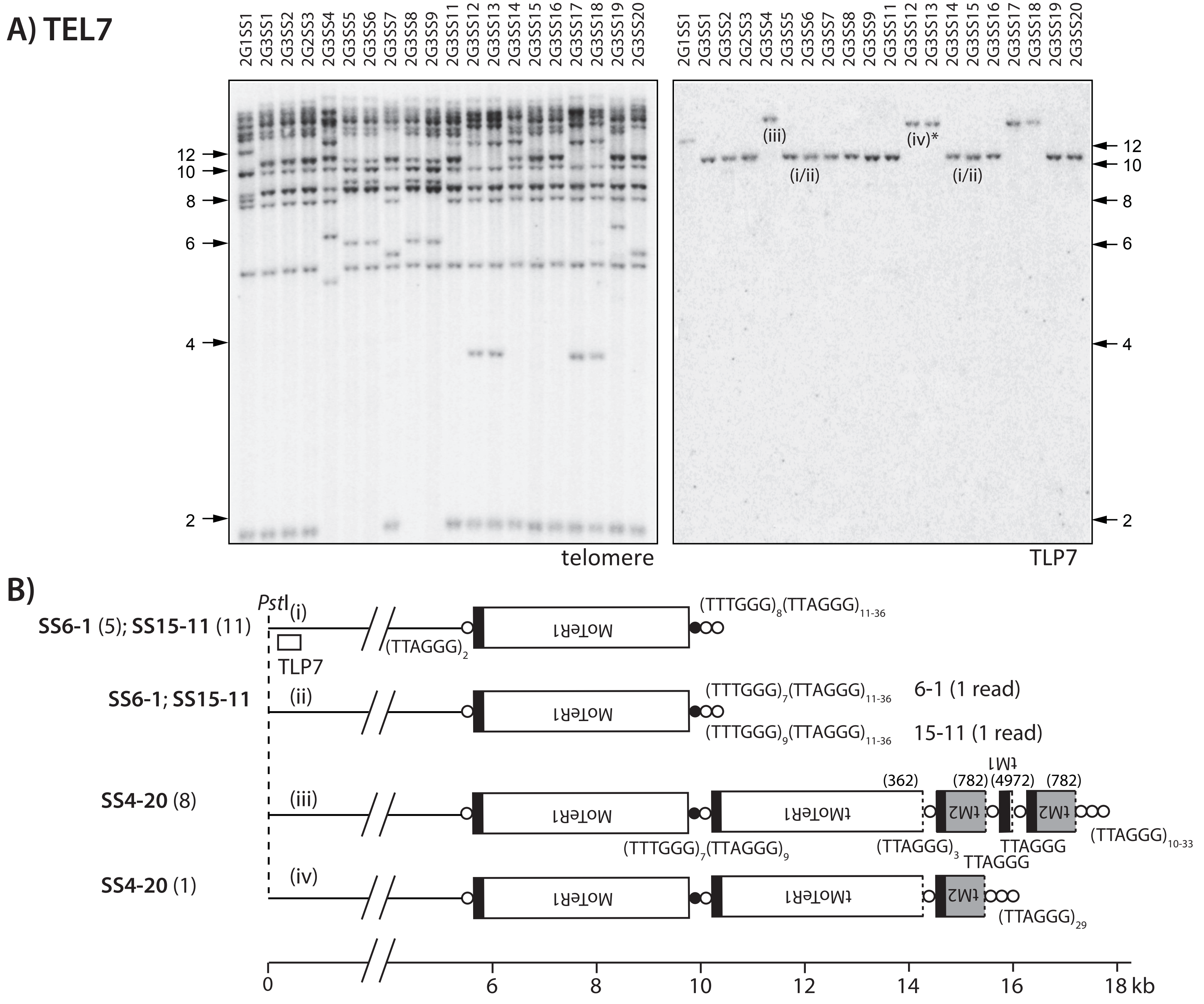


Figure S4. Rahnama_et_al.

TEL-D

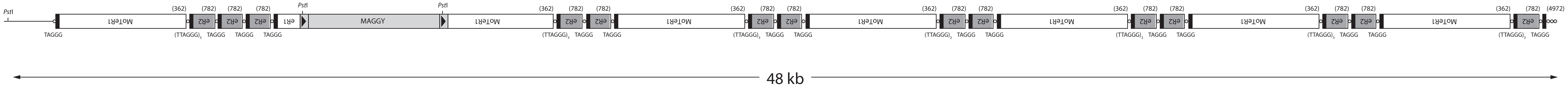


Figure S5. Rahnama et al.

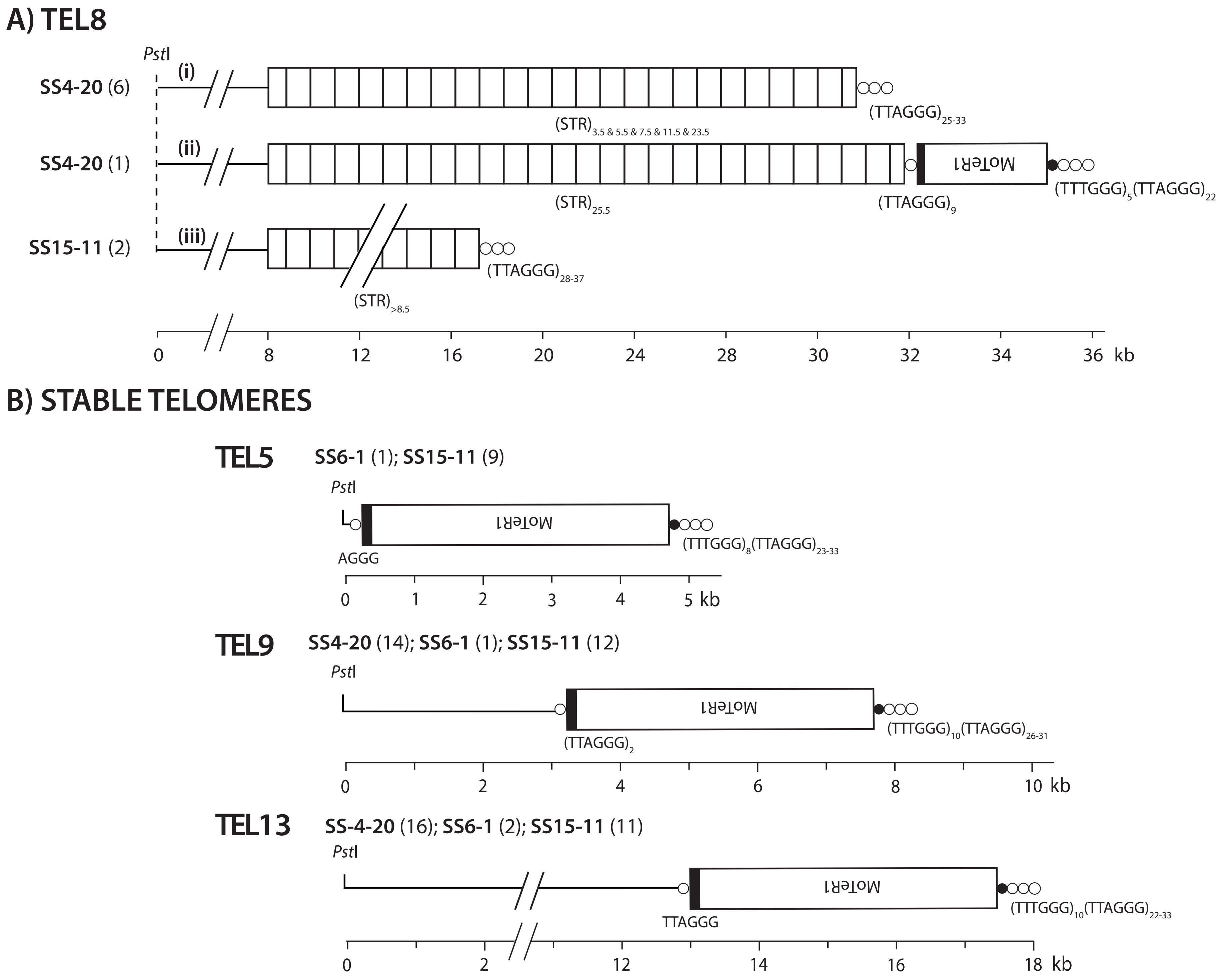


Figure S6. Rhanama et al.

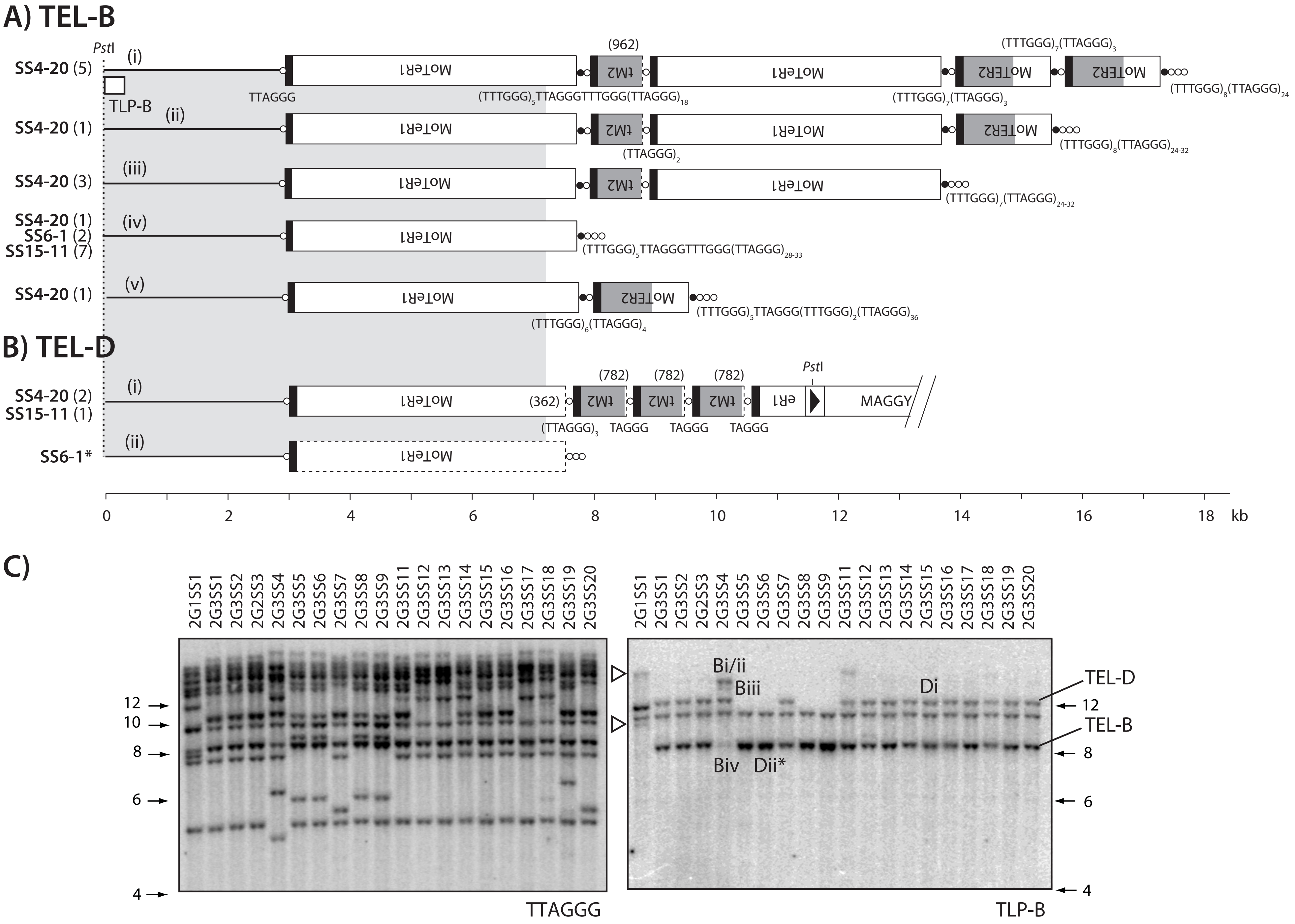


Figure S7. Rahnama et al.

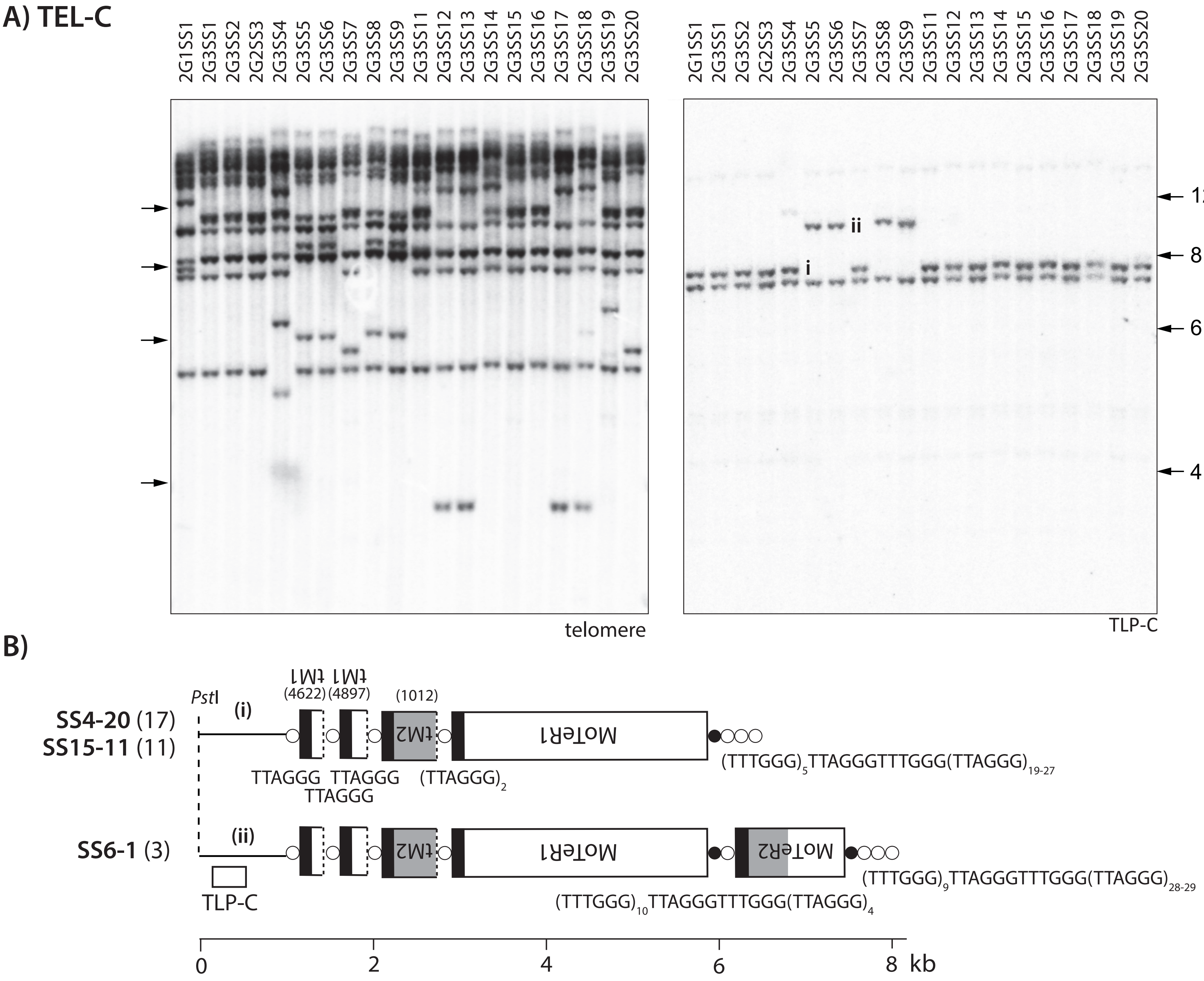


Figure S8. Rahnama et al.

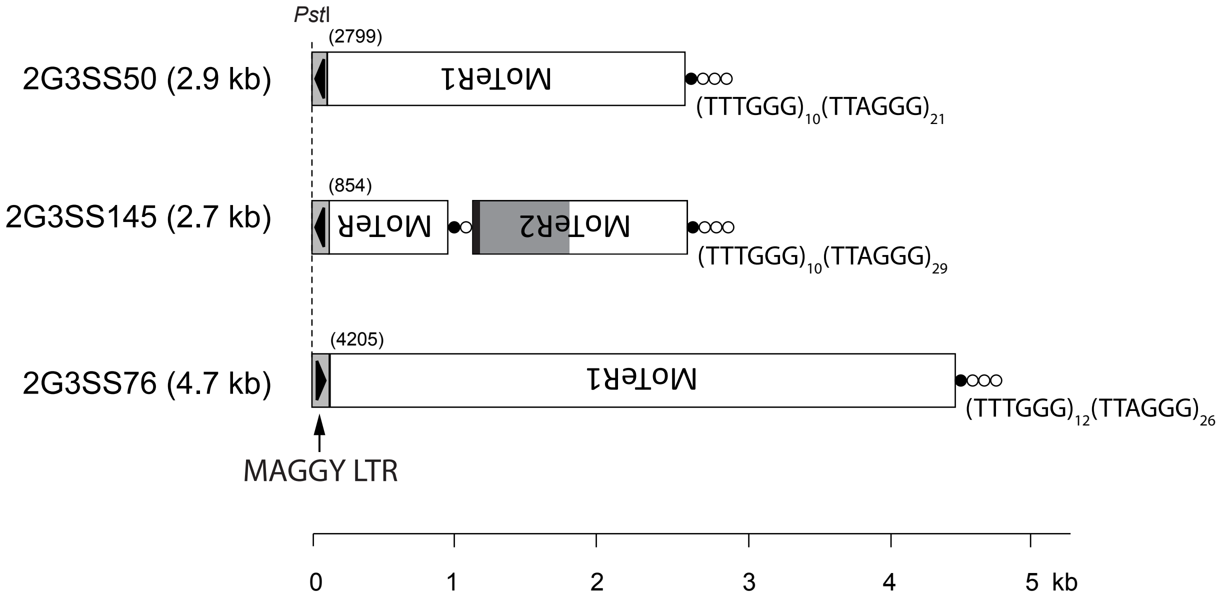


Figure S9. Rahnama et al.

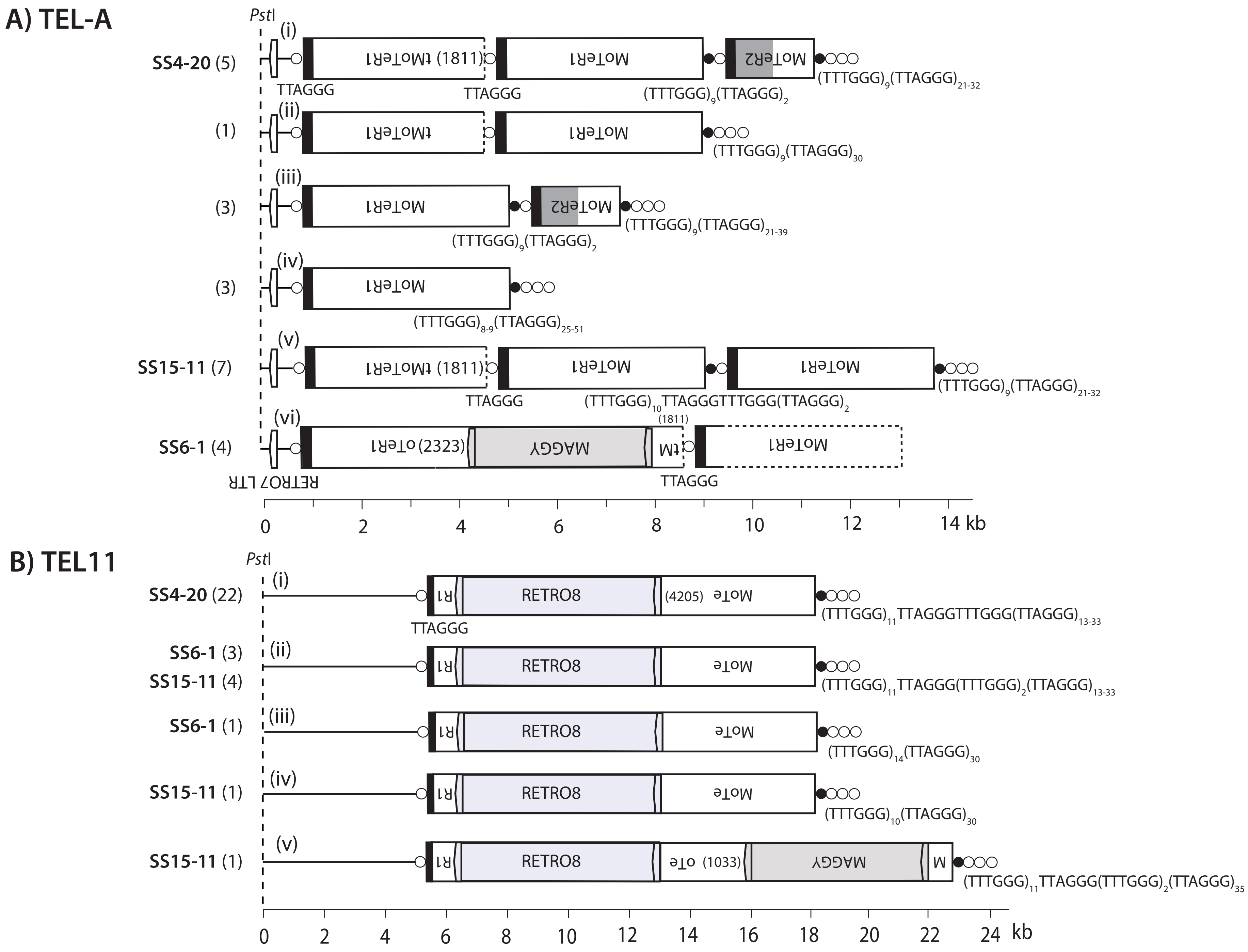


Figure S10_Rahnama_et_al.

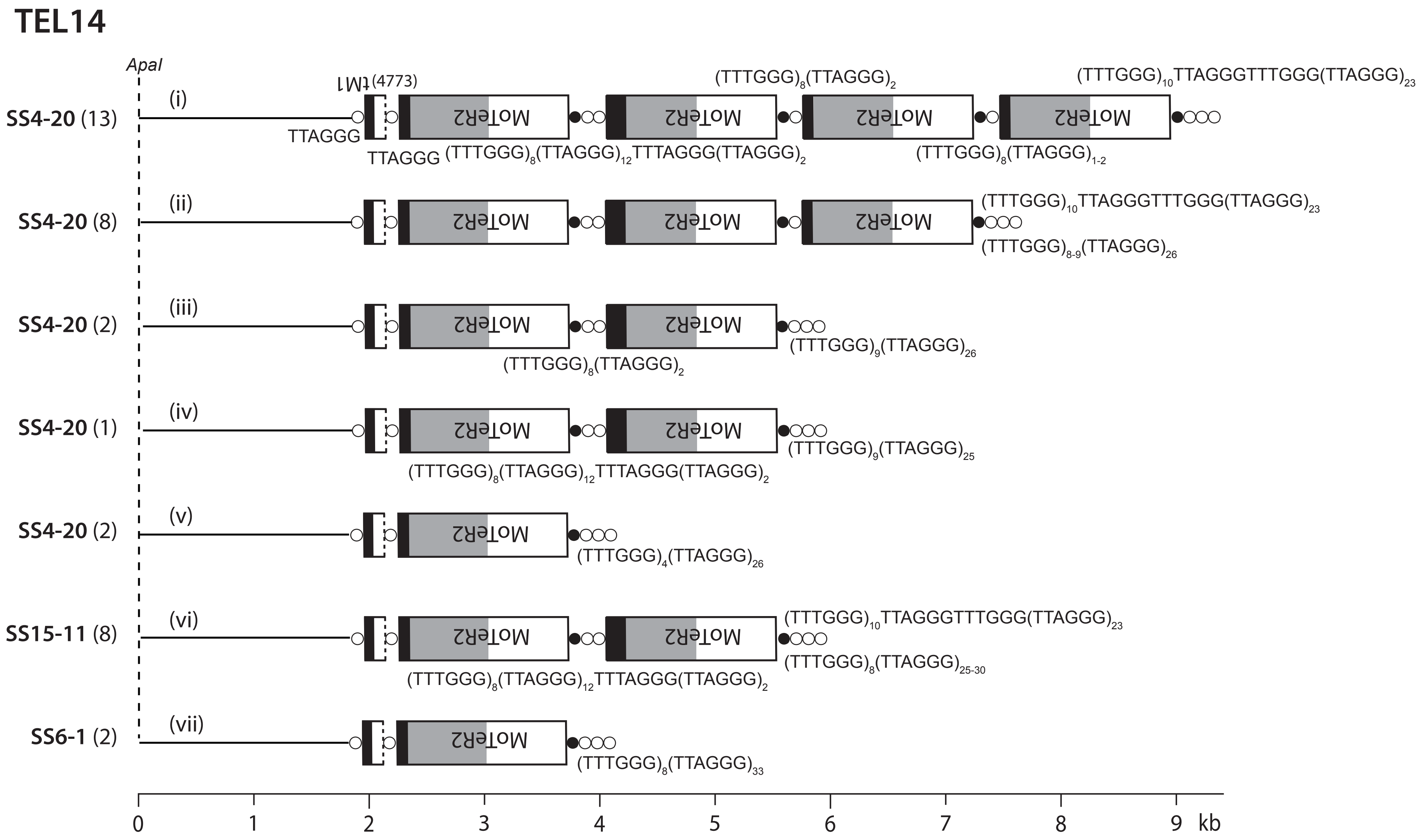


Figure S11. Rahnama et al.

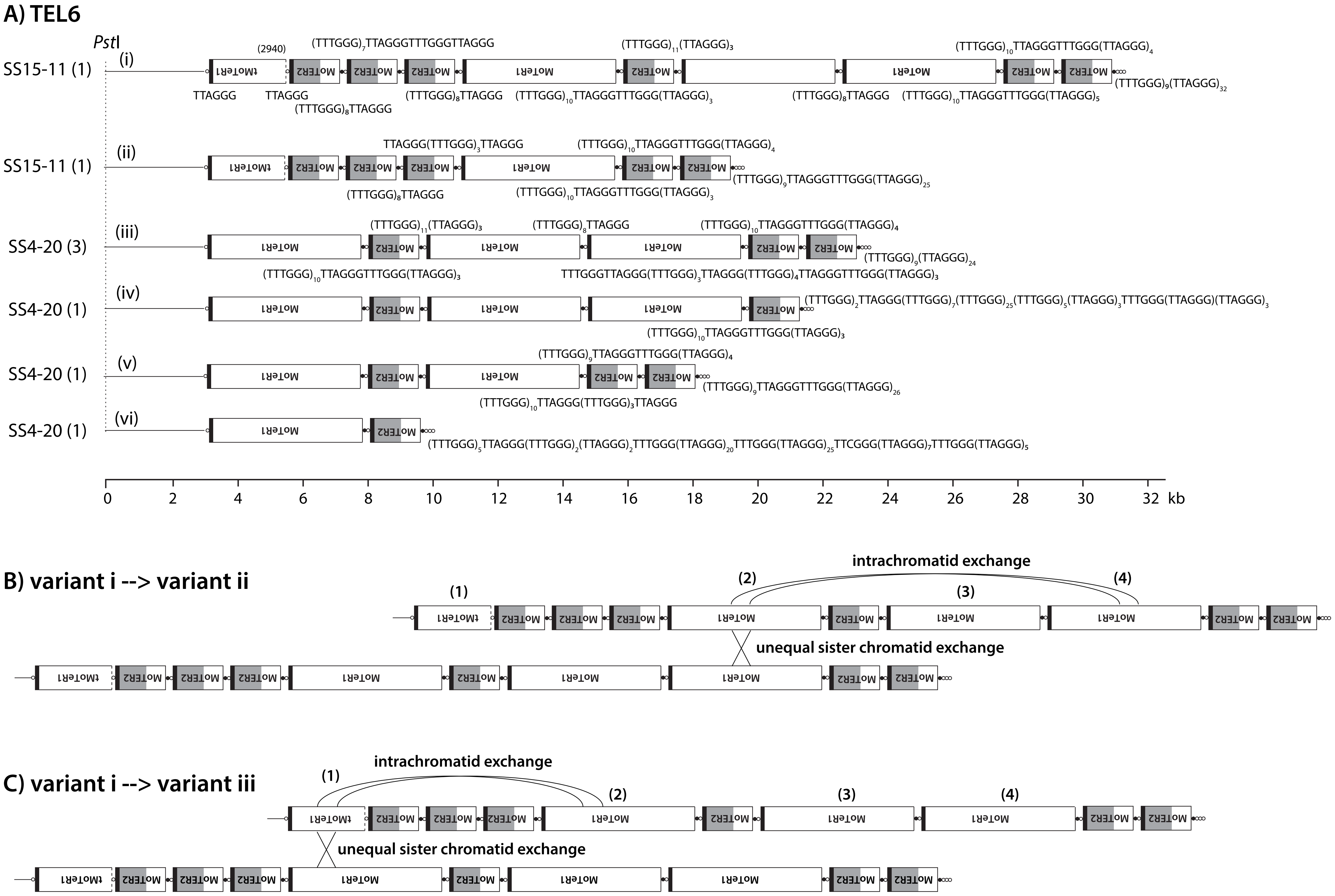


Figure S12. Rahnama et al.

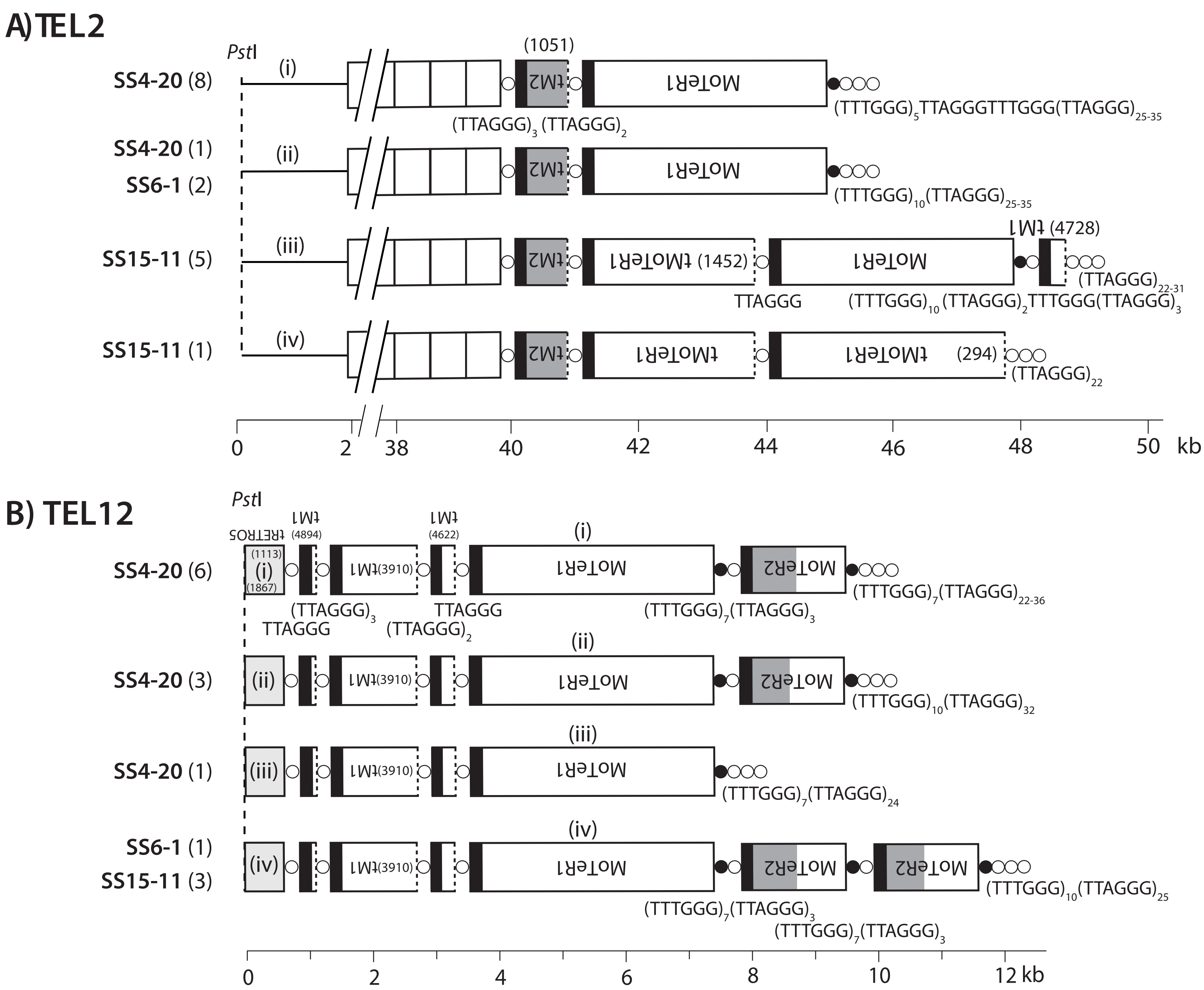


Figure S13. Rahnama_et_al.

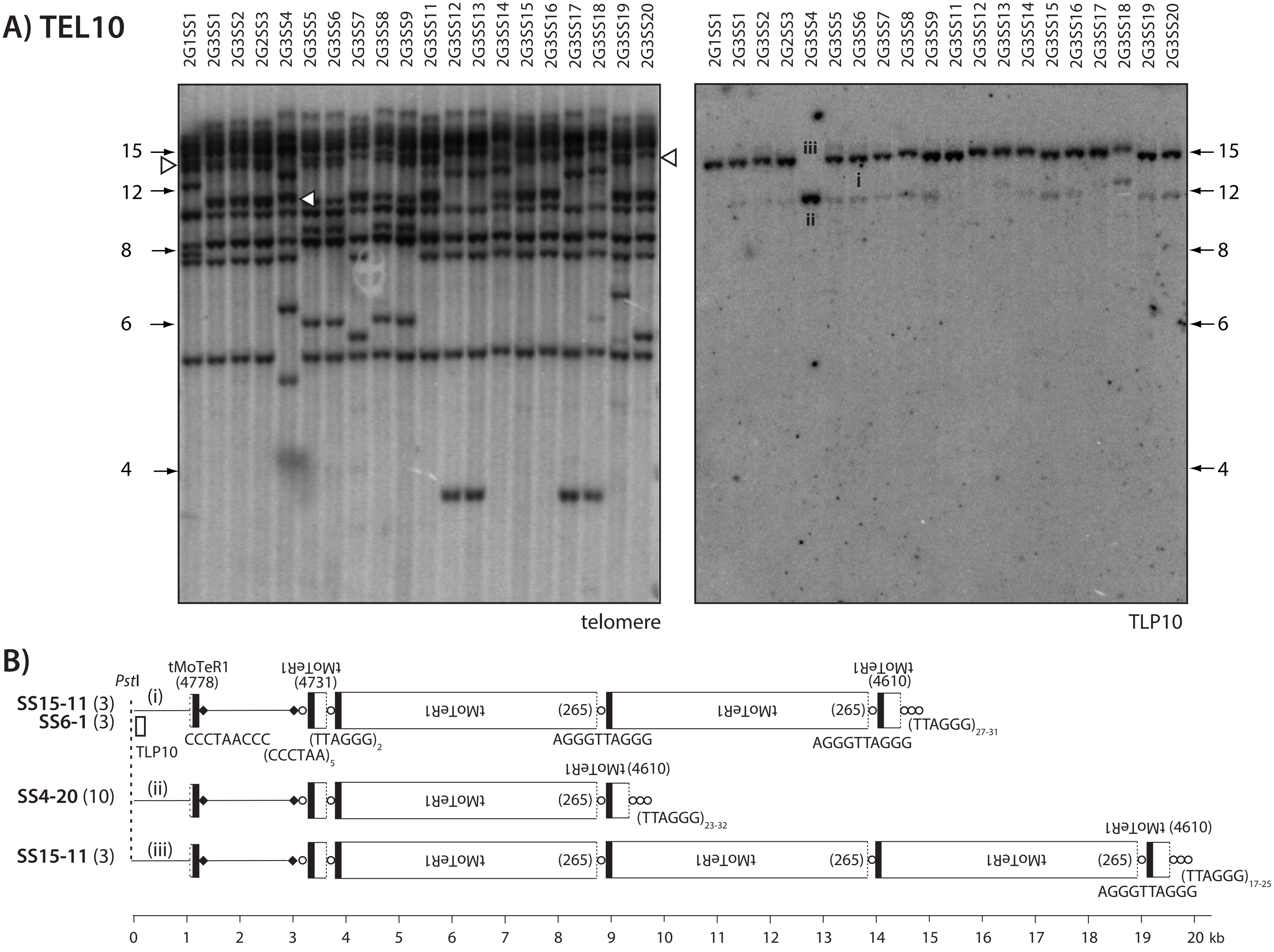


Figure S14. Rahnama et al.

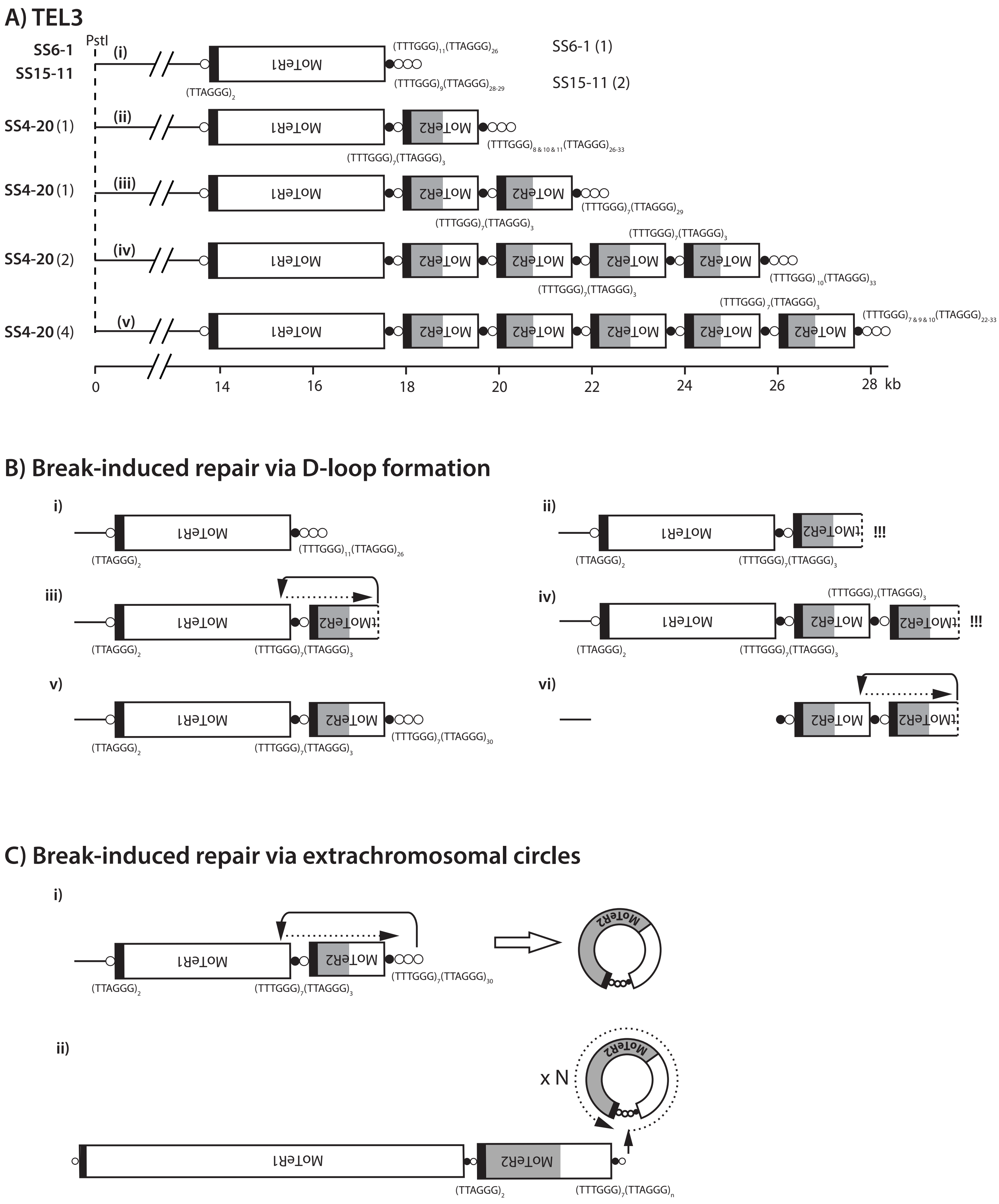


Figure S15. Rahnama et al.

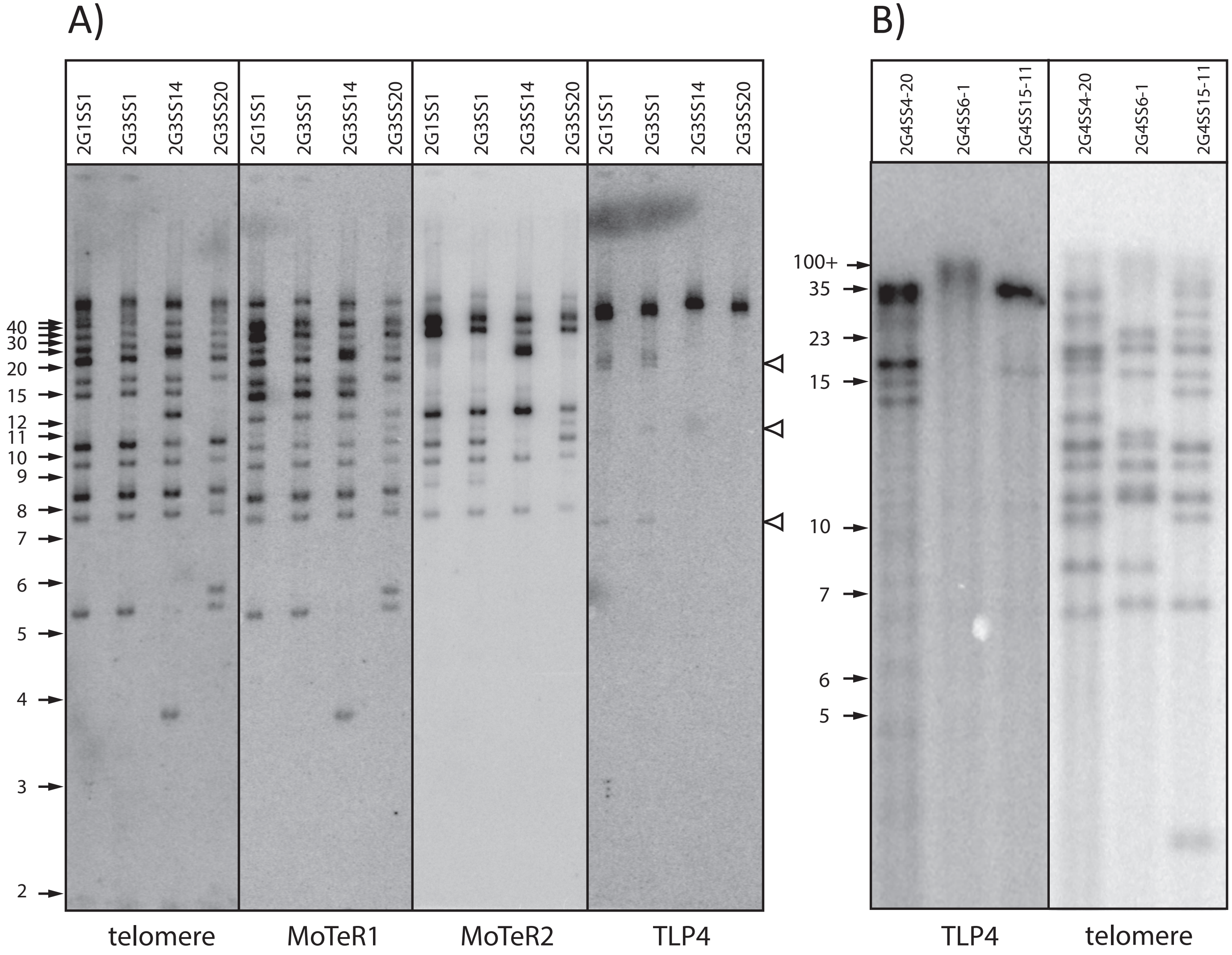


Figure S16. Rahnama et al.
